# Zinc Coordination Is Required for and Regulates Transcription Activation by Epstein-Barr Nuclear Antigen 1

**DOI:** 10.1371/journal.ppat.1000469

**Published:** 2009-06-12

**Authors:** Siddhesh Aras, Gyanendra Singh, Kenneth Johnston, Timothy Foster, Ashok Aiyar

**Affiliations:** 1 Department of Microbiology, Immunology & Parasitology, LSU Health Sciences Center, New Orleans, Louisiana, United States of America; 2 Stanley S. Scott Cancer Center, LSU Health Sciences Center, New Orleans, Louisiana, United States of America; 3 The Gene Therapy Program, LSU Health Sciences Center, New Orleans, Louisiana, United States of America; Emory University, United States of America

## Abstract

Epstein-Barr Nuclear Antigen 1 (EBNA1) is essential for Epstein-Barr virus to immortalize naïve B-cells. Upon binding a cluster of 20 cognate binding-sites termed the family of repeats, EBNA1 transactivates promoters for EBV genes that are required for immortalization. A small domain, termed UR1, that is 25 amino-acids in length, has been identified previously as essential for EBNA1 to activate transcription. In this study, we have elucidated how UR1 contributes to EBNA1's ability to transactivate. We show that zinc is necessary for EBNA1 to activate transcription, and that UR1 coordinates zinc through a pair of essential cysteines contained within it. UR1 dimerizes upon coordinating zinc, indicating that EBNA1 contains a second dimerization interface in its amino-terminus. There is a strong correlation between UR1-mediated dimerization and EBNA1's ability to transactivate cooperatively. Point mutants of EBNA1 that disrupt zinc coordination also prevent self-association, and do not activate transcription cooperatively. Further, we demonstrate that UR1 acts as a molecular sensor that regulates the ability of EBNA1 to activate transcription in response to changes in redox and oxygen partial pressure (pO_2_). Mild oxidative stress mimicking such environmental changes decreases EBNA1-dependent transcription in a lymphoblastoid cell-line. Coincident with a reduction in EBNA1-dependent transcription, reductions are observed in EBNA2 and LMP1 protein levels. Although these changes do not affect LCL survival, treated cells accumulate in G0/G1. These findings are discussed in the context of EBV latency in body compartments that differ strikingly in their pO_2_ and redox potential.

## Author Summary

Epstein-Barr virus (EBV) infects human B-cells and immortalizes them. Immortalization results in diseases that range from infectious mononucleosis to malignancies such as lymphomas. During immortalization, EBV expresses a small number of viral genes that modulate cellular proliferation and differentiation. One of the genes expressed by EBV, Epstein-Barr nuclear antigen 1 (EBNA1), activates the expression of the other viral genes required for immortalization. In this report, we have explored the mechanism by which EBNA1 activates gene expression. We have determined that EBNA1 uses the micronutrient zinc to self-associate, and that self-association is necessary for it to activate gene expression. Further, we have determined that environmental conditions such as oxygen tension and oxidative stress modulate EBNA1's capacity to self-associate, and therefore to activate gene expression. The gene expression profile and proliferative phenotype of EBV-infected cells is known to vary in differing environmental niches in the human body, such as lymph nodes and in peripheral circulation. We interpret our results to postulate that these differences arise as a consequence of varying oxygen tension in these microenvironments on EBNA1's capacity to activate viral gene expression. Our findings can be exploited to devise novel therapeutics against EBV-associated diseases that target EBNA1 through oxidative stress.

## Introduction

Epstein-Barr nuclear antigen 1 (EBNA1) has two functions that are necessary for Epstein-Barr virus (EBV) to immortalize naïve human B-cells. EBNA1 is essential for the replication and partitioning of EBV genomes in latently-infected cells [1], and activates the transcription of EBV genes that are essential for immortalization [2]. In earlier studies, the ability of EBNA1 to activate transcription was closely correlated to its ability to support EBV genome replication and partitioning during latency. Both activities require EBNA1 to bind a series of cognate binding sites termed the family of repeats (FR), and alterations in repeat number caused proportional variations in both functions [3].

There have been recent advances in understanding how EBNA1 activates the transcription from EBV promoters. It is known that occupancy of specific sequences by the chromatin boundary factor, CTCF, regulates EBNA1's ability to activate specific viral promoters during latency [4]. Additionally, studies have also distinguished the ability of EBNA1 to activate transcription from its ability to support the replication and partitioning of EBV genomes. The amino-terminal half of EBNA1 contains two positively-charged regions with alternating glycines and arginines that can bind AT-rich DNA in a manner similar to AT-hook proteins [5]. Indeed, the amino-terminal 450 amino-acids of EBNA1 can be replaced with a cellular AT-hook protein, HMGA1a, and the resulting chimera HMGA1a-DBD, supports replication and partitioning of *oriP*-plasmids and EBV genomes in transformed cell-lines [2],[6],[7]. Considering the role of HMGA1a in the formation of transcription enhanceosomes [8],[9], HMGA1a-DBD is surprisingly deficient in the ability to activate transcription from EBV promoters [2],[6]. These studies indicate that either AT-hook function is insufficient for transcription activation, or that it is irrelevant to the ability of EBNA1 to activate transcription.

Complementing the conclusions drawn using chimeric proteins such as HMGA1a-DBD, a 25 amino acid-long domain has been identified in the amino-terminus of EBNA that is required for EBNA1 to transactivate [10]. This domain, termed unique region 1 (UR1), is juxtaposed to the first of EBNA1's two AT-hooks. Deletion of UR1 does not abrogate EBNA1's ability to support the replication and partitioning of EBV-derived plasmids, but reduces its transactivation activity to the levels observed with HMGA1a-DBD [10]. Emphasizing the importance of UR1 in transactivation, addition of multiple copies of UR1 to HMGA1a-DBD restores its ability to activate transcription and facilitate the immortalization of naïve B-cells [2].

The mechanism by which UR1 contributes to EBNA1's ability to activate transcription is unknown. Here, we report that while UR1 is necessary for EBNA1 to transactivate, it is not sufficient for transactivation. We demonstrate that UR1 coordinates zinc via two essential cysteines residues. Chelation of cellular zinc selectively and significantly reduces the ability of EBNA1 to activate transcription. Consistent with these results, mutation of the essential cysteines, prevents the coordination of zinc *in vitro*, and also causes a transactivation defect *in vivo*. By co-immunoprecipitation and bimolecular fluorescence complementation, we show that UR1 can interact homotypically, and that this ability correlates strongly with EBNA1's ability to activate transcription. Further, when UR1 dimerizes by coordinating zinc, it facilitates interactions between dimers of EBNA1, and the latter are required for EBNA1 to transactivate cooperatively.

We demonstrate that the essential, conserved cysteines within UR1 are regulated by redox, and that the ability of EBNA1 to activate transcription is subject to the oxygen levels prevalent in the environment. At oxygen levels that mimic that found in lymph nodes, EBNA1 activates transcription from the EBV *Bam*HI-C promoter in a sustained manner; whereas at higher oxygen levels, transactivation is dampened rapidly. Exposure of a lymphoblastoid cell-line (LCL) to mild oxidative stress decreases transcription from the *Bam*HI-C promoter, a promoter active in latency III. Reductions are observed in the levels of EBNA2 and LMP1, genes whose promoters are transactivated by EBNA1 [11],[12],[13]. Although these changes do not affect the survival of treated cells, they accumulate in G0/G1. The results reported in this study provide the first molecular insights into the mechanism by which EBNA1 activates transcription, and describe environmental changes that modulate transactivation. We discuss these results in the context of gene-expression observed in proliferating EBV-immortalized cells in hypoxic environments such as the lymph node, and non-proliferating EBV-infected cells observed in the relatively oxygen-rich periphery.

## Results

### UR1 is necessary but not sufficient for transactivation by EBNA1

**Figure 1:**
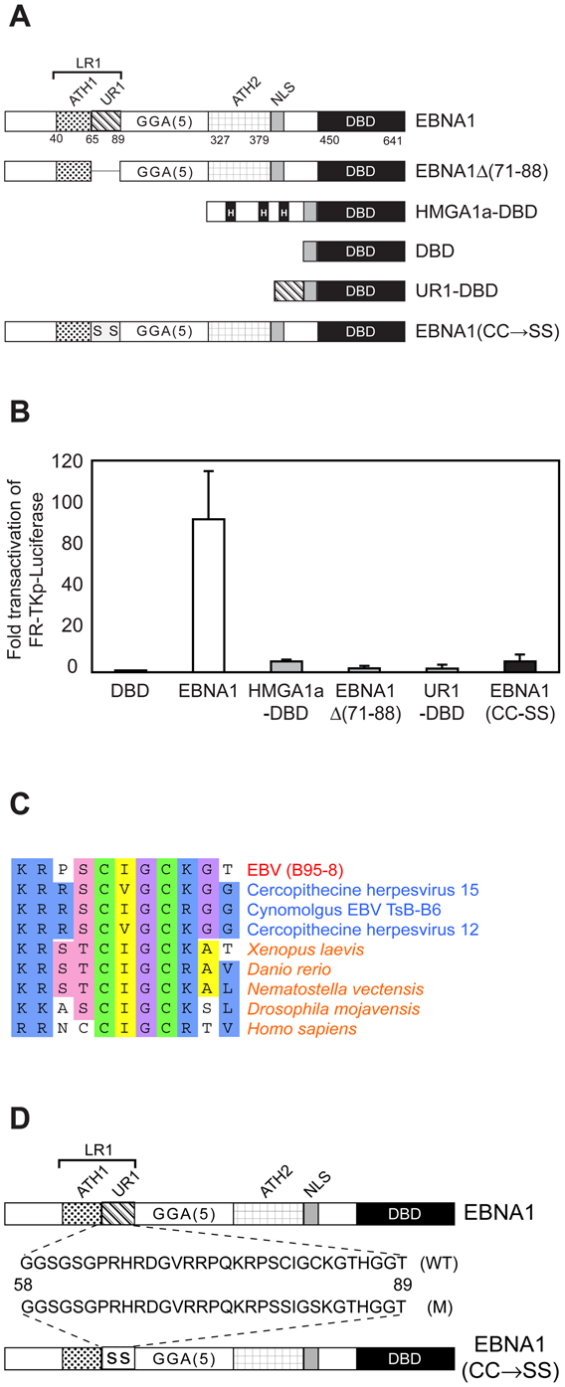
Two conserved cysteines in EBNA1's UR1 domain are essential for transactivation. (A) Schematic representation of the EBNA1 derivatives used in this study. The positions of the EBNA1's AT-hook domains (ATH1 & ATH2), UR1, DBD are indicated. The NLS spans from a.a 379–386. The EBNA1 derivatives used in this study contain a shortened glycine-alanine repeat that is 15 a.a. in length. EBNA1Δ(71-88) contains a deletion of a.a. 71–88 within the UR1 region. The HMGA1a-DBD protein contains the full length HMGA1a protein fused to the NLS, and the DBD. UR1-DBD contains a.a. 65–89 fused to a.a. 379–641 of EBNA1. The EBNA1(CC→SS) protein is described in detail below. (B) Transactivation of the FR-TKp-Luciferase reporter plasmid by the derivatives of EBNA1 shown in Figure 1A. C33a cells were individually co-transfected with the FR-TKp-Luciferase reporter plasmid, and expression plasmids for the indicated EBNA1 derivatives as described in the Methods section. Luciferase assays were performed at 48 hours post-transfection. Luciferase activity is expressed as fold over the level observed with when DBD is used as the effector protein. The data represents the average of at least three experiments for each EBNA1 derivative. (C) The UR1 region of EBNA1 is conserved in the EBNA1 orthologs of EBV-like gammaherpesviruses. Amino acids 75–85 of EBNA1 from EBV strain B95-8 (accession no. CA24816) (red) are aligned with the corresponding regions from EBNA1 proteins of other gammaherpesviruses (accession nos. YP_067973, BAB03281, AAA66373) (blue), and a portion of a C4 zinc-finger contained in the catalytic subunit of DNA polymerase δ from *Xenopus laevis* (accession no. NP_001087694), *Danio rerio* (accession no. CAM46996), *Nematostella vectensis* (accession no. XP_001641357), *Drosophila mojavensis* (accession no. XP_002008314), and *Homo sapiens* (accession no. P28340), identified by BLAST searches. Three or more alike amino-acids in the aligned sequences are shaded the same color. The Genbank accession number for each protein is indicated adjacent to it. (D) Schematic depiction of EBNA1 and the EBNA1(CC→SS) mutant, in which conserved cysteines at position 79 and 82 are mutated to serine. The sequences of wild-type (WT), and mutant (M) peptides used in this study are also shown.

EBNA1 augments transcription of reporter plasmids bearing the family of repeats (FR) by both retaining FR-containing DNAs within cells to increase the number of available transcriptional templates [10],[14], and by directly transactivating FR-dependent promoters [2],[6],[10]. While plasmid retention requires the AT-hook domains of EBNA1 [5], indicated as ATH1 and ATH2 in Figure 1A, an EBNA1 derivative with a deletion of a.a. 65–89 failed to activate transcription from a chromosomally integrated FR-dependent transcription reporter. The latter still contains ATH1 and ATH2, indicating that AT-hooks are irrelevant or insufficient for transcription activation. This conclusion is supported by results obtained using a chimera between the cellular AT-hook protein, HMGA1a, and the DBD of EBNA1. This chimera, HMGA1-DBD, retains *oriP*-plasmids as efficiently as EBNA1, but fails to activate transcription from the same chromosomally integrated transcription reporter [6]. HMGA1a-DBD activates transcription from transiently transfected FR-containing reporter plasmids with approximately 10% the efficiency of EBNA1 (*ibid*). Therefore, to distinguish the ability of EBNA1 derivatives to increase reporter transcription by plasmid retention versus transactivation, we have compared results obtained with wild type and mutant EBNA1 proteins to those obtained with HMGA1a-DBD (Figure 1B). In transient transfections of C33a cells, using FR-TKp-luciferase as the reporter plasmid, EBNA1 transactivates the TK-promoter approximately 85-fold over the EBNA1 DNA binding domain (DBD) alone. In contrast, a mutant of EBNA1 with a.a. 71–88 deleted, EBNA1Δ(71–88), activated transcription approximately two-times as well as the DBD alone. Using the same reporter, HMGA1a-DBD was observed to activate transcription approximately six-fold over the DBD. Having confirmed that the UR1 region of EBNA1 was necessary for transactivation to be observed even with episomal reporter plasmids, we tested if UR1 was sufficient for transactivation, using an UR1-DBD fusion protein. UR1-DBD failed to activate transcription from the FR-TKp-luciferase reporter, and was statistically indistinguishable from the effects of the DBD alone. Similar results were obtained upon transfection of EBV-negative BJAB Burkitt's lymphoma cells with *oriP*-*Bam*HI-Cp-luciferase used as the reporter (data not shown). Immunoblot analyses indicated that the failure of EBNA1Δ(71–88) and UR1-DBD to transactivate could not be attributed to lower expression levels or degradation (Figure S1). In this report, we have examined the mechanism by which UR1 facilitates transactivation by EBNA1.

Sequence comparison of the EBNA1 proteins from EBV and three related gammaherpesviruses indicated that a portion of UR1, corresponding to a.a. 75–85 of EBV's EBNA1 protein, was conserved in all four proteins (Figure 1C). A PSI-BLAST search conducted using these four conserved sequences matched portions of several zinc-binding proteins, particularly one-half of a conserved C4 zinc finger [15] present in the catalytic subunit of DNA polymerase δ from several species (Figure 1C). The matched sequences contain a pair of cysteine residues separated by two amino acids, which consist of an aliphatic amino acid and an invariant glycine. The cysteines are flanked by basic amino-acids, with an amino-acid whose side-chain contains a hydroxyl or sulfhydryl group immediately adjacent to the first cysteine. In light of the UR1's homology to half a zinc-binding domains, we tested whether the conserved cysteines residues in UR1 were necessary for EBNA1's ability to transactivate FR-dependent promoters. For this, both cysteines were altered to serines. A schematic of the mutant protein, EBNA1(CC→SS) is shown in Figure 1D, and by immunoblot analysis, EBNA1(CC→SS) was observed to be expressed as well as wild-type EBNA1 (Figure S2). Transactivation assays indicated that this mutant is severely impaired in its ability to activate transcription from the FR-TKp-luciferase reporter in C33a cells (Figure 1B) and *oriP*-*Bam*HI-Cp-luciferase reporter BJAB cells (data not shown).

In zinc-finger proteins, four cysteines, or a combination of four cysteines and histidines are used to coordinate zinc [15]. However, there are no other conserved cysteine or histidine residues present in the EBNA1 proteins from the four gammaherpesviruses, making it unlikely that these proteins coordinate zinc as monomers. Sequences that support multimerization were mapped previously to a fragment of EBNA1 containing UR1 [16]. Therefore, we postulated that the UR1 regions of adjacent EBNA1 molecules might coordinate zinc by contributing two cysteines apiece, and thereby dimerize. This was addressed experimentally by testing whether UR1 could coordinate zinc, and if zinc was required for EBNA1 to transactivate.

### Zinc is required for EBNA1 to activate transcription

**Figure 2:**
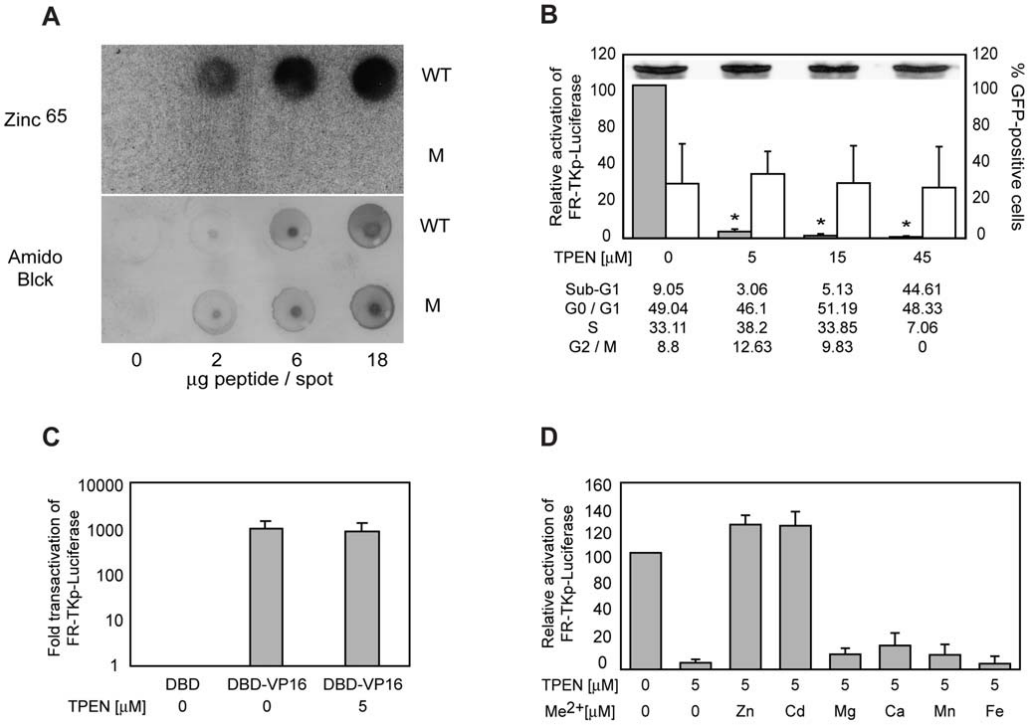
Zinc is coordinated by EBNA1's UR1 domain, and is required for EBNA1 to transactivate. (A) Evaluation of Zinc^65^ binding by the wild type (WT) and mutant (M) UR1 peptides. The indicated amounts of WT and M peptides were dot-blotted on a 0.2 mM PVDF membrane, and probed with radioactive zinc (∼50 µCi) as described in the Methods section. Washed membranes were dried and visualized using a Phosphorimager. WT peptide was observed to bind Zn^65^ in a concentration-dependent manner. In contrast, the M peptide failed to bind zinc. To confirm that both peptides bound the PVDF membrane, a blot prepared in parallel was dried and stained using Amido Black. (B) The metal chelator TPEN reduces the ability of EBNA1 to transactivate FR-TKp-Luciferase. C33a cells were co-transfected the FR-TKp-Luciferase reporter plasmid, an EBNA1-expression plasmid, and a CMV-EGFP expression plasmid. Cells were treated with the indicated levels of TPEN at the time of transfection, and harvested 15 hours later. Harvested cells were analyzed by flow cytometry to determine the fraction of live-transfected cells, the level of EGFP expression in that fraction. For cell-cycle analysis, the cells were fixed and then PI-stained. An aliquot of harvested cells were used to determine the expression level EBNA1 by immunoblot upon treatment with the indicated amounts of TPEN, shown as an inset in the graph. The rest of the cells were processed to determine luciferase activity, which is expressed as a percent of the luciferase activity observed in the absence of TPEN treatment, and shown in the grey bars. The open bars indicate the percent of live EGFP-positive cells at each concentration of TPEN. The asterisks indicate that the significantly lower levels of luciferase activity were observed in the presence of 5, 15 and 45 µM TPEN (p<0.05, Wilcoxon rank-sum test). The cell-cycle profile of TPEN-treated and control cells was obtained for one experiment, and is shown below the graph. (C) TPEN does not affect transactivation by DBD-VP16. C33a cells were co-transfected with the FR-TKp-Luciferase reporter plasmid, and expression plasmids for DBD, and DBD-VP16. Some DBD-VP16 transfected cells were treated with the indicated amounts of TPEN at the time of transfection, and analyzed 15 hours later to determine the fraction of live-transfected cells, and the level of luciferase expression. The activity observed with DBD-VP16 in the absence and presence of TPEN is expressed as the fold-over the luciferase level observed with DBD alone. (D) Zinc and Cadmium reverse TPEN-mediation inhibition of transactivation by EBNA1. C33a cells were co-transfected with the FR-TKp-Luciferase reporter plasmid, an EBNA1 expression plasmid, and a CMV-EGFP expression plasmid, and treated with 5 µM TPEN at the time of transfection. Fifteen hours post-TPEN addition, 5 µM of Zn (CH_3_COO)_2_, Cd(CH_3_COO)_2_, CaCl_2_, MgSO_4_, MnCl_2_, or Fe(CH_3_COO)_2_, was added to the cells for an additional 15 hours. Cells were harvested and analyzed by flow cytometry to determine the level of live-transfected cells, followed by determination of luciferase activity. The relative activation is shown in the grey bars, and is expressed as a percent of the luciferase activity observed in the untreated sample.

To determine if UR1 coordinates zinc, peptides corresponding to a.a 58–89 of EBNA1 and EBNA1(CC→SS), indicated as WT and M in Figure 1D, were tested for their ability to bind radioactive zinc by a zinc-blot procedure previously used to identify and characterize several proteins that bind zinc [17],[18],[19]. As shown in Figure 2A, the wild-type peptide, but not the mutant, was observed to bind Zn^65^. Parallel blots were stained with amido-black to confirm that both peptides bound PVDF with similar efficiencies.

To test whether zinc was necessary for EBNA1 to transactivate, transfected cells were exposed to TPEN, a chelator known to have high specificity for zinc. Previously, TPEN has been used to probe the ability of p53 and other proteins to bind zinc within cells [20],[21],[22]. As shown in Figure 2B, TPEN diminished transactivation by EBNA1 in dose-dependent manner, such that exposure to 5 µM TPEN for 15 hours reduced transactivation by EBNA1 to approximately 4% of the untreated control. In contrast, a higher level of TPEN, approximately 40 µM, was required to affect the function of p53 [21]. EBNA1's ability to transactivate was also reduced in the presence of 1.5 µM and 3 µM TPEN (data not shown). Because exposure to TPEN for shorter or similar lengths of time has been shown to induce apoptosis in some cell-lines [20], a cell-cycle analysis was performed on TPEN-treated transfected C33a cells, and is summarized in Figure 2B. C33a cells were more resistant to TPEN-induced apoptosis than HeLa cells, BJAB cells and lymphoblastoid cell lines (data not shown). At 45 µM TPEN, a substantial sub-G1 peak was observed. In contrast, at lower concentrations of TPEN the cell-cycle profiles of TPEN-treated cells were similar to those observed for untreated cells. Immunoblot analysis indicated that TPEN did not decrease transactivation by inhibiting the synthesis, or inducing the degradation of EBNA1 (Figure 2B). The effect of TPEN was also examined on a co-transfected CMV-EGFP reporter plasmid, and found to not significantly change the fraction of live, GFP-positive cells, or the mean fluorescence index of these cells. To investigate the possibility that transcription from the TK promoter require other zinc-dependent transcription factors affected by TPEN, we examined whether TPEN altered transactivation by a chimeric protein in which the DBD was fused to the acidic activation domain of VP16 [23] (DBD-VP16). DBD-VP16, which lacks the N-terminal 450 a.a of EBNA1, activates transcription from the FR-TKp-luciferase reporter (Figure 2C), and this activation is not reduced by treatment with 5 µM TPEN. Thus, exposure to TPEN specifically reduces the ability of EBNA1 to transactivate the FR-dependent transcription reporter plasmids. Next, we tested whether inhibition by TPEN was reversible by the subsequent addition of zinc or other metal ions (Figure 2D). Transfected cells were treated with 5 µM TPEN for 15 hours, following which zinc or other metal ions were added for an additional 15 hours prior to analysis. It was observed that addition of Zn^2+^ or Cd^2+^ effectively restored transactivation, in contrast to Mg^2+^, Ca^2+^, Mn^2+^ and Fe^2+^ (Figure 2D). Addition of Zn^2+^ or Cd^2+^ did not affect basal transcription from the FR-TKp-luciferase reporter, or transactivation of this reporter by DBD-VP16 (data not shown). The ability of zinc to restore EBNA1-dependent transactivation in TPEN-treated cells was concentration dependent, and did not affect expression from a co-transfected CMV-EGFP reporter (Figure S3).

### The amino-terminal 450 amino acids of EBNA1 do not squelch transactivation by EBNA1

**Figure 3:**
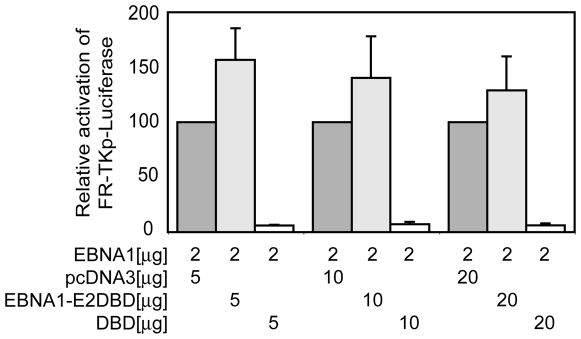
EBNA1(1-450)-E2DBD does not squelch transactivation by EBNA1. C33a cells were co-transfected with the FR-TKp-Luciferase reporter plasmid, and control vector pcDNA3 (dark grey bars), an expression plasmid for EBNA1(1-450)-E2DBD (light grey bars), or an expression plasmid for DBD (open bars, in the amounts indicated below each bar. Cells were harvested at 48 hours post-transfection, normalized by flow cytometry for the number of live-transfected cells, and analyzed for luciferase activity, which is expressed as percent luciferase activity relative to the activity obtained by co-transfection with the same amount pcDNA3.

We initially postulated that UR1 might facilitate a zinc-dependent interaction between EBNA1 and a cellular transcription co-activator. To identify such proteins, a yeast two-hybrid screen was performed using the first 94 amino acids of wild-type EBNA1 as the bait protein against a cDNA library from HeLa cells, a cell-line in which EBNA1 has been observed to activate transcription. No protein identified in this screen associated specifically with the bait protein, but not an UR1-deleted derivative (data not shown). In an effort to test our hypothesis that UR1 associates with cellular transcription factors, we tested whether co-expression of EBNA1's amino-terminal 450 a.a. interferes with EBNA1's ability to activate transcription from an FR-dependent reporter plasmid, by squelching its interaction with putative cellular co-activators or general transcription factors [24],[25]. As shown in Figure 3, co-transfecting increasing amounts of an expression plasmid encoding a fusion between the first 450 a.a. of EBNA1 and the DNA binding domain of the BPV-1 E2 protein, 3xF-EBNA1(1-450)-E2DBD, did not affect the ability of EBNA1 to activate transcription from the FR-TKp-luciferase reporter, over the pcDNA3 control. In contrast, co-transfecting a DBD expression plasmid decreased the expression observed from FR-TKp-luciferase, by expressing a protein that competes efficiently with EBNA1's ability to bind FR [26]. While 3xF-EBNA1(1-450)-E2DBD does not interfere with the function of wild-type EBNA1, it retains the ability to activate transcription from a TKp-luciferase reporter plasmid containing twenty E2-binding sites (Figure S4), and this transactivation is sensitive to treatment with 5 µM TPEN (Figure S5). Thus, the inability of 3xF-EBNA1(1-450)-E2DBD to squelch transactivation of FR-TKp-luciferase by EBNA1 is not because it is incapable of activating transcription via a mechanism similar to that used by EBNA1.

Some proteins transactivate by directly altering DNA template structure. For example, the prototypic cellular AT-hook protein, HMGA1a, bends DNA to mediate transcription enhanceosome formation [8],[27]. HMGA1a, p53, *Ikaros*, and other proteins are also known to homotypically interact, loop intervening DNA, and thereby juxtapose a distal enhancer to a promoter [28],[29],[30]. EBNA1 has AT-hooks, and is known to loop *oriP*-DNA [31],[32]. Looping activity correlates to domains in the first 450 a.a. of EBNA1, particularly UR1 [16],[33],[34]. Therefore, we tested whether UR1 functions as a zinc-dependent self-association domain.

### UR1 is a zinc-dependent self-association domain

**Figure 4:**
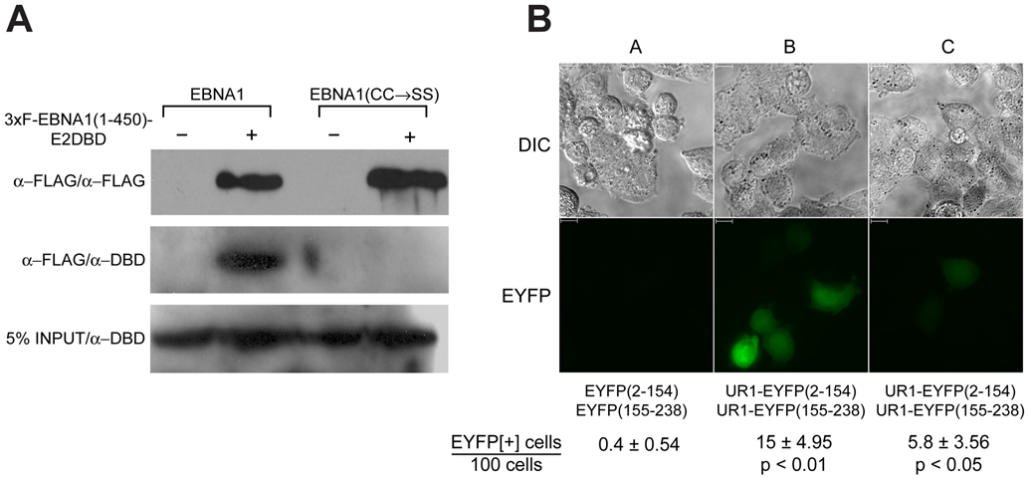
EBNA1 can self-associate through its UR1 domain. (A) The amino-terminal 450 amino-acids of EBNA1 can self-associate. 293 cells were co-transfected with expression vectors for 3xF-EBNA1(1-450)-E2DBD, and either EBNA1 or EBNA1(CC→SS). 48 hours post-transfection, lysates were prepared from harvested cells. EBNA1(1-450)-E2DBD was immunoprecipitated using the M2 monoclonal anti-FLAG antibody conjugated to sepharose beads. Immunoprecipitates were separated on an 8% SDS-PAGE gel, transferred to PVDF membranes and immunoblotted using M2 mouse monoclonal anti-FLAG antibody (α-FLAG/α-FLAG), or rabbit polyclonal antibodies directed the EBNA1 DBD (α-FLAG/α-DBD). 5% of the lysates were directly immunoblotted for EBNA1 or EBNA1(CC→SS) (5% lysate/α-DBD). (B) Self-association of UR1 can be detected by bimolecular fluorescence complementation: 293 cells were transfected with expression plasmids for (A) EYFP(2-154) and EYFP(155-238), or (B) & (C) expression plasmids for UR1-EYFP(2-154) and UR1(155-38). Transfected cells were treated with (B) vehicle, or (C) 80 µM 1,10-phenanthroline. 48 hours after transfection, cells were visualized by fluorescence for EYFP (YFP) or by light microscopy (DIC). The scale bars indicate a length of 10 microns. The fraction of fluorescent cells observed under each condition in five independent measurements is shown along with the standard deviation. The number of fluorescent cells observed with the UR1-containing EYFP derivatives is significantly greater than the number seen with the EYFP derivatives by themselves (*p*<0.01). A significant decrease in the number of florescent cells is observed after treatment with 1,10-phenanthroline (*p*<0.05) when compared to the untreated cells.

Co-immunoprecipitation was used to test if EBNA1(1-450)-E2DBD associates with wild-type EBNA1, and EBNA1(CC→SS). For this, EBNA1(1-450)-E2DBD, which is amino-terminally tagged with a 3xFLAG epitope, was co-expressed in 293 cells along with EBNA1 or EBNA1(CC→SS). Lysates were incubated with a monoclonal anti-Flag antibody linked to sepharose beads, and examined by immunoblot. As shown in Figure 4A, EBNA1 was co-precipitated with EBNA1(1-450)-E2DBD using the anti-FLAG antibody. In contrast, EBNA1(CC→SS) was never observed to associate with EBNA1(1-450)-E2DBD. Immunoblots using anti-EBNA1 antibody indicated that EBNA1 and EBNA1(CC→SS) were expressed equivalently in lysates.

Having determined that the amino-terminal 450 a.a. of EBNA1 could self-associate if the sequence of UR1 was not altered, we tested whether UR1 was sufficient to mediate homotypic interactions using bimolecular fluorescence complementation [35]. For this, UR1 was fused to amino acids 2-154 of EYFP, or to amino acids 155-238 of EYFP. Expression plasmids were co-transfected into 293 cells, and examined by fluorescence microscopy. While no fluorescence was observed using the parental plasmids expressing both halves of EYFP (Figure 4B), or with each of the UR1 plasmids transfected singly (data not shown), the UR1 fusions complemented each other (Figure 4B). The ability of zinc chelators to disrupt complementation was tested using 1,10-phenanthroline, which has been used widely to dissect the role of zinc association with various proteins [36],[37]. TPEN could not be used for this experiment because even low levels of TPEN cause 293 cells to dissociate from the coverslip. Transfected cells were treated with 80 µM 1,10-phenanthroline for 24 hours, and then examined by microscopy. Treatment of transfected cells with 80 µM 1,10-phenanthroline for 24 hours caused a large decrease in the number and intensity of EYFP-positive cells (Figure 4B). These results indicate that UR1 can self-associate in a manner dependent on metal ions.

### The nature of self-association mediated by UR1

**Figure 5:**
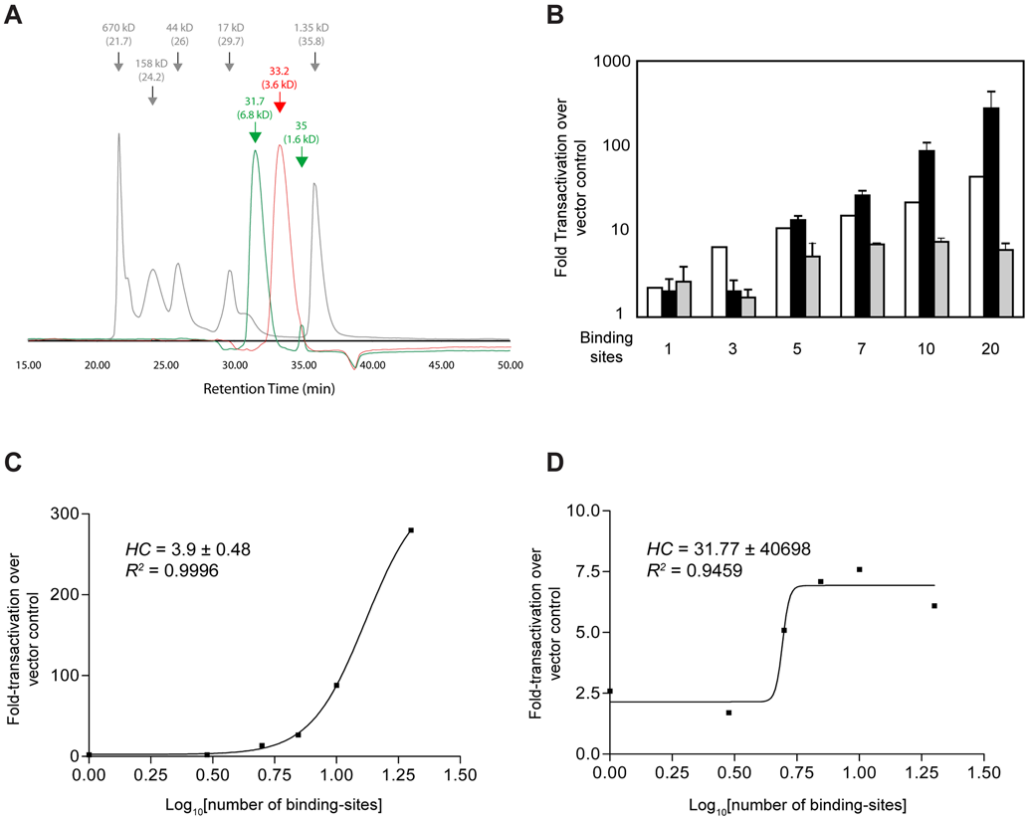
UR1-mediated self-interaction is required for EBNA1 to transactivate cooperatively. (A) Size-exclusion chromatography of the wild-type UR1 peptide indicates zinc-dependent dimerization. WT peptide was subjected to HPLC size-exclusion chromatography, as described in the Methods section, in the absence of zinc (red), or in the presence of 1 mM zinc sulfate in the buffer (green). The retention time is indicated above each peak, and the apparent molecular weight calculated from the retention time is shown in parentheses. The elution profile of molecular weight markers is shown in gray. The known molecular weight of each marker is indicated above the peak, with the observed retention time shown in parentheses. (B) Cooperative transactivation by EBNA1 is UR1-dependent. C33a cells were co-transfected individually with TKp-luciferase reporter plasmids containing one, three, five, seven, ten, or 20 EBNA1 binding sites in FR along with an expression plasmid for EBNA1 or EBNA1(CC→SS). Cells were harvested at 48 hours post-transfection, normalized by flow cytometry for the number of live-transfected cells, and analyzed for luciferase activity, which is expressed as luciferase activity fold-over the activity obtained by co-transfection with vector control. The white bars indicate the predicted levels of transactivation for EBNA1 if it increased additively with increasing numbers of binding sites. The transactivation observed with EBNA1 is indicated by the black bars, and transactivation observed with EBNA1(CC→SS) is indicated by the gray bars. For reporter plasmids containing up to three binding sites, EBNA1 and EBNA1(CC→SS) transactivated equivalently. A small difference was observed for a reporter plasmid with five binding sites, and this difference was greatly accentuated for reporter plasmids containing seven, ten, and 20 binding sites. (C) Cooperative transactivation by EBNA1 fits a sigmoidal dose-response model by non-linear regression analysis. The fold transactivation by EBNA1 observed in (5B) was analyzed for cooperativity as described in the Materials & Methods, and found to fit a sigmoidal dose-response model well, as indicated by the goodness of fit (R^2^). Positive-cooperativity is inferred from the positive Hill coefficient (HC). (D) EBNA1(CC→SS) does not activate transcription cooperatively. The fold transactivation observed for EBNA1(CC→SS) was fit to the same sigmoidal dose-response model used in 5C. The low R^2^ indicates that transactivation by EBNA1(CC→SS) poorly fits this model. In addition, the large standard deviation observed for the HC also indicates a lack of cooperative transactivation.

While the previous results indicate that UR1 can mediate dimerization as evaluated by bimolecular fluorescence complementation, they do not distinguish between direct association and indirect association mediated by a cellular protein. If zinc coordination results in direct association, it is relevant to consider that in addition to forming dimers, pairs of cysteines can form trimers and tetramers, as observed in the GAL4 DBD [38], or in the RING finger [39]. To distinguish between such multimeric forms, we examined the migration properties of the wild-type UR1 peptide in the presence and absence of zinc by size exclusion chromatography. After establishing conditions to observe a highly reproducible elution profile for molecular weight standards, the peptides were examined by chromatography in the presence and absence of 1 mM zinc sulfate. The results of this analysis are shown in Figure 5A. The apparent molecular weight (MW) calculated from the peak retention time for the peptides in the absence of zinc were close to their theoretical MWs. WT peptide eluted as a single peak in the absence of zinc with an apparent molecular weight of 3.6 kD (Figure 5A). When the wild-type peptide was analyzed in the presence of zinc, two peaks were observed: 1) a small peak, representing approximately 5% of the total, migrated at an apparent molecular weight of 1.6 kD; 2) a large peak, consistent with size of a dimer that migrated at a apparent molecular weight of 6.8 kD. The presence or absence of zinc had no effect on the retention time of the mutant peptide (data not shown). These data indicate that at least in the context of a peptide, UR1 dimerizes in the presence of zinc.

The ability of UR1 to dimerize indicates that either EBNA1 contains a second intra-molecular dimerization domain at its N-terminus, or that dimers of EBNA1 may form larger inter-molecular complexes through UR1. To distinguish between these two possibilities, attempts were made using cross-linkers or Blue native electrophoresis to resolve complexes formed by wild-type EBNA1 within transfected cells. In such gels, we could not distinguish between multimers of EBNA1 dimers, and EBNA1 associated with cellular proteins that interact with EBNA1, such as p32/TAP/gC1qR [40], Importin-α/Rch1 [41], p40/EBP2 [42], and Brd4 [43] (data not shown).

Therefore, we resorted to an alternative approach in an effort to distinguish between intra-molecular and inter-molecular dimerization mediated by UR1. This approach was chosen on the basis of two alternative hypotheses for the mechanism by which UR1 dimerization may contribute to transactivation. In the first, it was hypothesized that UR1 was necessary to form intra-molecular dimers that are essential for transactivation. Alternatively, it was hypothesized that inter-molecular dimerization by UR1 was necessary to form a structured array of EBNA1 bound to FR, and that such arrays were necessary for transactivation. These two hypotheses can be distinguished by examining how transactivation by wild-type EBNA1 or EBNA1(CC→SS) varies with the number of EBNA1-binding sites. If UR1 is necessary to form intra-molecular dimers required for transactivation, then it is predicted that EBNA1(CC→SS) will transactivate poorly relative to wild-type EBNA1, independent of the number of binding sites. In contrast, if UR1 is necessary to form inter-molecular dimers required for transactivation, then it is predicted that for a single binding site or a small number of binding sites, EBNA1(CC→SS) will transactivate equivalently to EBNA1, with a defect in transactivation being exposed as the number of binding sites is increased. Therefore, the ability of wild-type EBNA1 and EBNA1(CC→SS) to transactivate a series of reporter plasmids containing one, three, five, seven, ten and 20 binding-sites was examined under conditions previously determined to be optimal to observe cooperative transactivation (Figure 5B).

The white bars in Figure 5B indicate the level of transactivation predicted with increases in the number of binding sites, assuming a simple additive relationship between the two. In contrast, as shown in Figure 5B, and observed previously [3],[44], transactivation by EBNA1 (black bars) is strongly cooperative, with a synergism that is proportional to the number of binding sites. Under optimal conditions, EBNA1 bound to 20 binding-sites transactivates approximately 150-fold over EBNA1 bound to a single binding-site, and it is important to note that the latter is reproducibly two to three-fold over what is observed in the absence of EBNA1. Transactivation by EBNA1(CC→SS) (gray bars) could not be distinguished statistically from wild-type EBNA1 for reporter plasmids containing one, three or five binding sites. We note that for such small numbers of binding sites, wild-type EBNA1 does not display significant synergism. In contrast to wild-type EBNA1, at higher numbers of binding sites, EBNA1(CC→SS) does not transactivate cooperatively. We interpret these results to indicate that the major contribution of UR1 toward EBNA1's ability to transactivate is by facilitating cooperativity; the latter being more consistent with inter-molecular interactions than intra-molecular dimerization.

When the correlation between transactivation and number of binding sites is fit to a sigmoidal dose-response curve by non-linear regression, strong positive cooperativity is observed for wild-type EBNA1, with a Hill coefficient (HC) of 3.9±0.48 (Figure 5C), and a goodness of fit (R^2^) of 0.9996. No cooperativity is observed when transactivation by EBNA1(CC→SS) is fit to the same sigmoidal dose-response curve (HC = 31.77±40698), and the data fit this model poorly (R^2^ = 0.9459) (Figure 5D).

In conjunction with other results presented here (Figure 4 and Figure 5A), it is concluded that UR1 facilitates cooperative transactivation by mediating inter-molecular interactions between adjacent EBNA1 dimers bound to FR. It remains to be determined whether such cooperative interactions facilitate architectural changes at the promoter, or association with a coactivator. We note that if a multimerization-dependent coactivator mediates transactivation, it is equally inefficient in associating with wild-type EBNA1 and EBNA1(CC→SS) when these proteins are bound to one, three or five binding-sites.

While our data favor a role for UR1 in inter-molecular associations over intra-molecular dimerization, we reiterate that either interaction remains dependent on the ability of two cysteines residues within UR1 to coordinate zinc. The ability of cysteines to associate with zinc is regulated by their redox status. Reduced cysteines can coordinate zinc, whereas oxidized cysteines cannot. For zinc-finger proteins, and other transcription factors, it has been observed that the redox state of cysteine modulates activity.

### Redox modulates EBNA1's ability to transactivate

**Figure 6:**
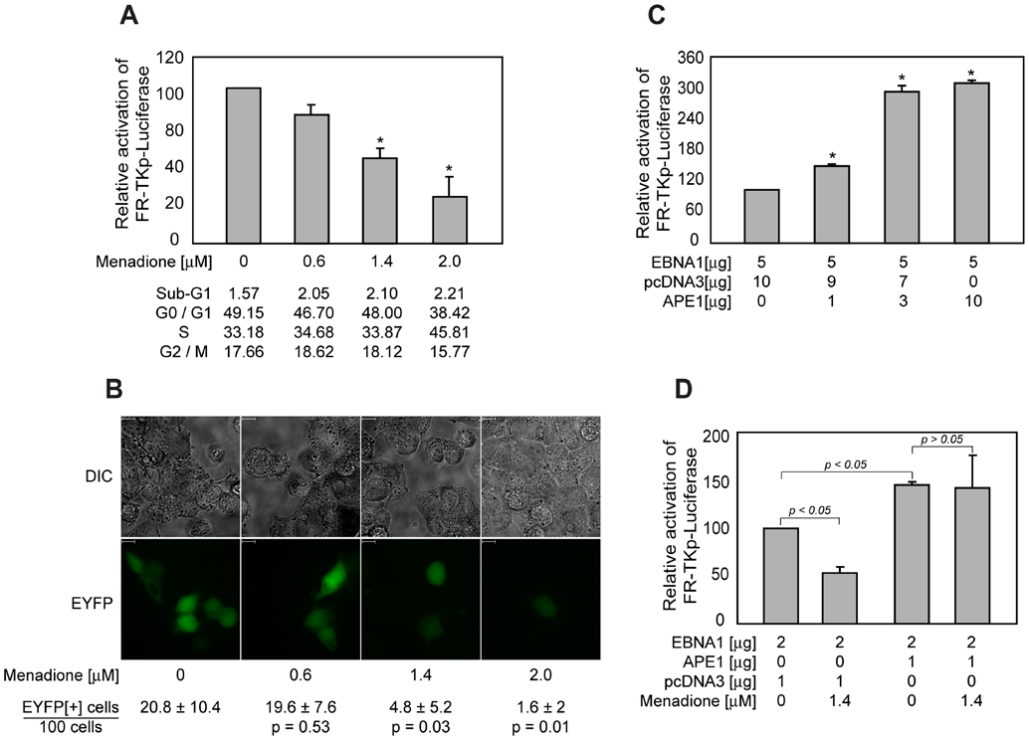
Transactivation by EBNA1 is sensitive to oxidative stress, and is augmented by over-expression of APE1/Ref-1. (A) Menadione reduces the ability of EBNA1 to transactivate FR-TKp-Luciferase. C33a cells were co-transfected the FR-TKp-Luciferase reporter plasmid, and an EBNA1-expression plasmid. Cells were treated with the indicated levels of menadione six hours post-transfection, and harvested 18 hours later. For cell-cycle analysis, an aliquot of cells was fixed and then PI-stained. The rest of the cells were processed to determine luciferase activity, which is expressed as a percent of the luciferase activity observed in the absence of menadione treatment, and shown in the grey bars. The cell-cycle profiles of menadione-treated and control cells were obtained for one experiment, and are shown below the graph. The asterisks indicate statistical significance (*p*<0.05) compared to control. (B) Menadione affects the ability of UR1 to support bimolecular fluorescence complementation. C33a cells were transfected with expression plasmids for A) EYFP(2-154) and EYFP(155-238), or B) & C) expression plasmids for UR1-EYFP(2-154) and UR1(155-238). Transfected cells were treated with vehicle alone, or 0.6, 1.4 and 2.0 µM menadione six hours after transfection. Cells were visualized by fluorescence for EYFP (YFP) or by light microscopy (DIC) after 18 hours of menadione treatment. The scale bars indicate a length of 10 microns. The fraction of fluorescent cells observed for each menadione concentration in five independent measurements is shown along with the standard deviation. The *p*-values indicate statistical significances calculated using the Wilcoxon rank-sum test upon a pair-wise comparison to untreated cells. *p*-values lower than 0.05 indicate significance. (C) Over-expression of Ref-1/APE1 augments transactivation by EBNA1. C33a cells were transfected with an EBNA1 expression plasmid, and increasing amounts of an expression plasmid for Ref-1/APE1 along with the FR-TKp-luciferase reporter plasmid. Luciferase levels were evaluated 48 hours post-transfection, and are expressed as a fraction of transactivation observed in the absence of Ref-1/APE1, which was set to be 100%. The asterisks indicate statistical significance (*p*<0.05) compared to control. (D) Over-expression of Ref-1/APE1 ameliorates the effect of menadione on EBNA1 mediated transactivation. C33a cells were transfected with an EBNA1 expression plasmid, and either 1 µg of a Ref-1/APE1 expression plasmid or empty vector control plasmid in addition to the FR-TKp-luciferase reporter plasmid. Six hours post-transfection, the cells were split and half the cells were treated with 1.4 µM of menadione. Luciferase levels were evaluated 24 hours post-transfection. *p* values are indicated in the figure.

Transactivation by AP-1, NFκB, and zinc-binding proteins such as p53 and Egr-1 is modulated by intracellular redox, and is increased two to three-fold by over-expression of redox factor 1 (Ref-1/APE1) [45],[46],[47],[48],[49]. The oxidation status of zinc-coordinating cysteines regulates the activity of several zinc-finger transcription factors, such as Sp1, Egr-1 and steroid hormone receptors [45],[50],[51],[52], because oxidation of sulfhydryl groups (-SH) results in the progressive generation of sulfenic acid (-SOH), sulfinic acid (-SO_2_H) and sulfonic acid (-SO_3_H), none of which can coordinate zinc. Therefore cysteine oxidation alters the structure, and consequentially the function of zinc-binding proteins [53]. Menadione, a redox-reactive *p*-quinone has been used to study the effect of redox on proteins containing reactive cysteines, including estrogen receptor and the SMN complex [54],[55]. Because zinc coordination is required for EBNA1 to activate transcription, we tested whether exposure to menadione affected transactivation. C33a cells co-transfected with an EBNA1-expression plasmid and the FR-TKp-luciferase reporter plasmid were split six hours post-transfection and exposed to increasing levels of menadione for 18 hours prior to analysis. Menadione reduced transactivation in a dose-dependent manner (Figure 6A), without affecting expression of a co-transfected CMV-EGFP reporter plasmid (data not shown). Although transactivation was reduced to as little as 20%, no profound change in the cell-cycle profile of transfected cells was observed upon menadione treatment at concentrations up to 2 µM (Figure 6A). The highest concentration of menadione used here is considerably lower than concentrations of menadione used to induce apoptosis via oxidative stress [56]. A similar 50% decrease in transactivation was observed upon treatment of transfected cells with 150 µM of the mild oxidant paraquat (Figure S6). Menadione did not affect transactivation by DBD-VP16 from the same reporter (Figure S7), indicating that it is unlikely to have inactivated components of the basal transcription apparatus used at the HSV-1 Tk promoter. Bimolecular fluorescence complementation was used to examine whether oxidative stress impaired the ability of UR1 to support dimerization, which is essential for transactivation. Cells transfected with the UR1-EYFP derivatives described above were split six hours post-transfection, and then exposed to increasing concentrations of menadione for 18 hours prior to analysis by fluorescence microscopy. At concentrations of menadione above 0.6 µM, a statistically significant reduction in the fraction of fluorescent cells was observed (Figure 6B), with a concomitant decrease in fluorescence intensity (Figure 6B and data not shown). In the absence of menadione, approximately 20 fluorescent cells were observed for every 100 adherent cells. In the presence of 1.4 µM menadione, where a 50% reduction in transactivation was observed (Figure 6A), approximately five fluorescent cells were observed for every 100 adherent cells. Together, these results indicate that the reduction in transactivation observed upon oxidative stress correlates with an impaired ability of UR1 to support dimerization.

During oxidative stress, thioredoxin directly reduces cysteines on target transactivators, or increases the amount of reduced Ref-1/APE1 available to reduce cysteines on target proteins [57],[58]. One characteristic of cysteines that are reduced by Ref-1/APE1 is their close juxtaposition to lysine and/or arginine residues. The conserved cysteines in UR1 lie in such a connotation (Figure 1C). It was therefore tested whether over-expression of Ref-1/APE1 would affect EBNA1's ability to transactivate. For this, C33a cells were co-transfected with an EBNA1-expression plasmid, the FR-TKp-luciferase reporter plasmid, and increasing amounts of an expression plasmid for Ref-1/APE1. This analysis, shown in Figure 6C, indicated that over-expression of Ref-1/APE1 increased the ability of EBNA1 to activate transcription by as much as 3-fold, a magnitude of increase similar to its effect on other redox-sensitive transcription factors [45],[46],[47],[48],[49]. Ref-1/APE1 did not increase transactivation by DBD-VP16 from the same reporter, and did not affect expression of either DBD-VP16 or EBNA1 (data not shown). Because Ref-1/APE1 augmented EBNA1's ability to transactivate, we tested whether over-expression of Ref-1/APE1 would ameliorate the effect of oxidative stress induced by menadione. For this, C33a cells were transfected with an EBNA1-expression plasmid, the FR-TKp-luciferase reporter plasmid and either an APE1-expression plasmid, or empty vector control. Cells were split six hours post-transfection, and one-half treated with 1.4 µM menadione. As is shown in Figure 6D, cells that over-express Ref-1/APE1 are resistant to menadione treatment. Similarly, over-expression of Ref-1/APE1 reversed the effect of 150 µM of paraquat on EBNA1 (Figure S8). Thus, as reported for several other viral and cellular transactivators, Ref-1/APE1 also facilitates EBNA1's ability to transactivate by modulating redox.

### Transactivation by EBNA1 is sustained under hypoxic conditions

**Figure 7:**
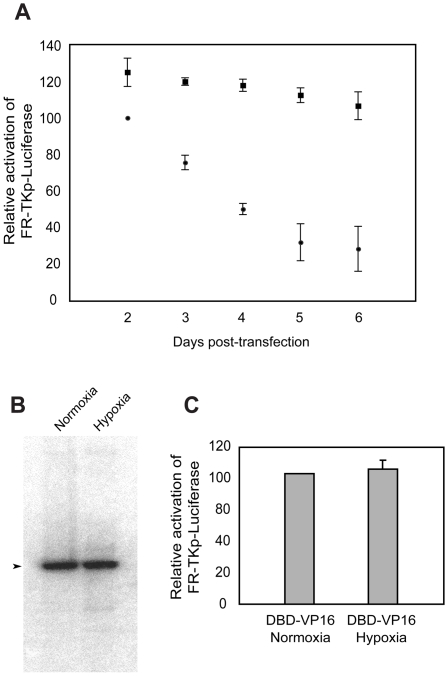
Hypoxic conditions prolong EBNA1's capacity to transactivate. (A) Transactivation by EBNA1 is sustained under hypoxic conditions. C33a cells were co-transfected with an EBNA1-*oriP* expression plasmid and the *oriP*-*Bam*HI-Cp-luciferase reporter plasmid. Transfected cells were split six hours post-transfection, and grown under normoxic (filled circles) or hypoxic conditions (filled squares). Luciferase expression was measured at the indicated time-points, and is expressed relative to the luciferase expression observed under normoxic conditions 48 hours post-transfection. (B) Oxygen levels do not affect *oriP*-plasmid replication and maintenance. A Southern blot was performed to examine the levels of replicated (*Dpn*I-resistant) *oriP*-*Bam*HI-Cp-luciferase plasmid recovered after six days under normoxic or hypoxic conditions. The arrowhead indicates replicated, full-length *oriP*-*Bam*HI-Cp-luciferase. (C) Environmental oxygen levels do not alter the activity of DBD-VP16. C33a cells were transfected with a DBD-VP16 expression plasmid and the FR-TKp-luciferase reporter plasmid. Luciferase activity was measured three days post-transfection in cells exposed to normoxic or hypoxic conditions.

B-cells infected latently by EBV reside in the periphery and internal body compartments such as lymph nodes and tonsilar tissue. These compartments differ considerably in the availability of O_2_, so that the oxygen partial pressure (pO_2_) is approximately 80 torr (10.5% O_2_) in the periphery while the pO_2_ in lymph ranges between 20–40 torr (2.5%–5% O_2_) [59],[60], or lower [61]. The latently infected cells in these compartments are dramatically different, with distinct patterns of viral gene expression. Cells in the lymph node express all the EBV genes necessary to drive proliferation of latently-infected cells (latency III). In contrast, latently-infected cells in the periphery are largely quiescent, and do not express any viral proteins, with the exception of EBNA1 when cells divide (latency 0/I) [62],[63]. While it is clear that there are epigenetic changes on the EBV genome associated with these two distinct patterns of latency [4],[64],[65], the contributions of viral proteins toward this “imprinting” is unknown. Transactivators, such as NFκB, Egr-1, p53 and AP-1, whose activities are increased by over-expression of Ref-1/APE1, are also more active under hypoxic conditions [47],[66],[67],[68]. We therefore tested the effect of hypoxia on EBNA1's ability to transactivate. For this, C33a cells were co-transfected with an EBNA1-*oriP* expression plasmid, and the *oriP*-*Bam*HI-Cp-luciferase reporter plasmid. Transfected cells were split six hours post-transfection, and incubated in parallel under normoxic (21% O_2_) and hypoxic (4% O_2_) conditions for six days, and it is emphasized that the latter mimic the pO_2_ found in lymph nodes [59],[60],[61]. The expression of luciferase was measured daily during this time-course, and the amount of replicated *oriP*-*Bam*HI-Cp-luciferase reporter plasmid was measured at the end of six days. At the earliest measurement point, 48 hours post-transfection, the levels of luciferase were approximately 25% higher in cells maintained under hypoxia than cells maintained in normoxic conditions (Figure 7A). For both conditions, the level of luciferase expression decreased over time; however, the rate of decrease was more rapid under normoxic conditions than hypoxic conditions. As a consequence of the difference in rates, by six days post-transfection, the average amount of luciferase expressed in hypoxic cells was 3.5-fold the level expressed in normoxic cells (Figure 7A). Southern blots performed to quantify the level of replicated *oriP*-*Bam*HI-Cp-luciferase reporter plasmids six days post transfection, indicated equivalent numbers of plasmids per cell under both conditions (Figure 7B). The latter result indicates that differences in luciferase expression between hypoxic and normoxic conditions cannot be attributed to differences in EBNA1's association with its cognate binding sites in *oriP* under these two conditions. To confirm this, an expression plasmid for DBD-VP16 was co-transfected with FR-TKp-luciferase into C33a cells that were subsequently maintained at hypoxic or normoxic conditions for 72 hours. No difference in luciferase expression was detected (Figure 7C), reiterating the conclusion that the difference in luciferase activity under normoxic and hypoxic reflects a difference in EBNA1's ability to activate transcription under these two conditions, and not its ability to bind FR or support *oriP* replication and maintenance. These experiments provide evidence that UR1 can act as a molecular sensor that modulates EBNA1's ability to activate transcription in response to environmental conditions.

To determine the relevance of these observations for EBV gene-expression within immortalized cells, we examined the effects of oxidative stress on transcription from the *Bam*HI-C promoter in a LCL.

### The effect of oxidative stress on *Bam*HI-Cp transcription in a lymphoblastoid cell-line

**Figure 8:**
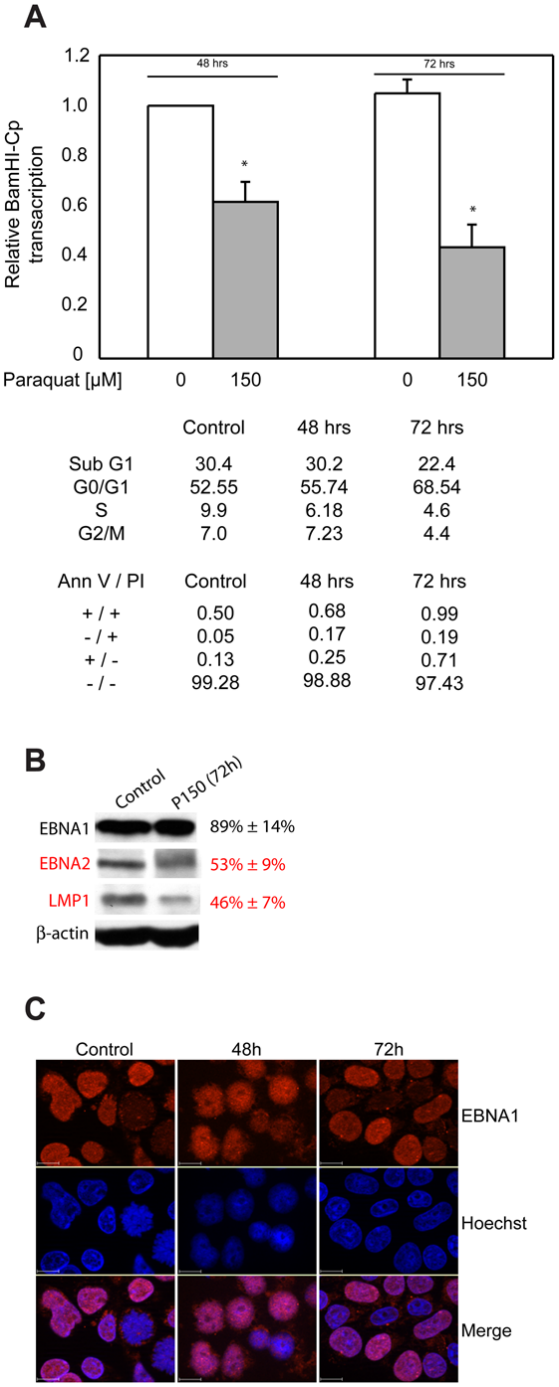
Mild oxidative stress reduces transcription from the EBV *Bam*HI-Cp in a lymphoblastoid cell-line. (A) Paraquat treatment reduces the level of *Bam*HI-Cp transcripts in treated NOLA-1 cells. Total RNA was extracted from control or cells exposed to 150 µM of paraquat for 48 or 72, reverse-transcribed with random hexamers. cDNA was used for real time PCR performed as described in the Methods section to evaluate changes in Cp transcripts relative to the endogenous control GAPDH transcripts. The results are presented relative to the *Bam*HI-Cp transcript level observed in control cells, which was assigned a value of 1. Significant decreases in the level of *Bam*HI-Cp transcripts were observed in cells with paraquat for 48 and 72 hours, such that transcript level was reduced to approximately 60% after 48 hours of treatment, and approximately 40% after 72 hours of treatment. Cell-cycle analyses conducted with several treated samples indicates that paraquat treatment does not increase the sub-G1 population. However, treated cells were consistently observed to accumulate in G0/G1. Treatment caused a small increase in the number of necrotic cells doubly-positive for annexin V and PI staining. Small increases were also seen for cells positive for only annexin V or only PI. (B) Paraquat treatment decreases expression of EBNA2 and LMP1, as evaluated by immunoblot. 2×10^5^ control or cells treated with 150 µM paraquat (P150) for 72 hours were examined as described in Methods for expression of EBNA1, EBNA2 and LMP1. Band intensity was compared to that observed in control cells, and is expressed as percent of control along with standard deviation from two experiments. Expression was normalized to the level of β-actin protein detected in each sample. (C) Paraquat treatment does not alter the expression or localization of EBNA1. Control or treated cells were transferred to slides by cytospin, prior to fixation and permeabilization. EBNA1 expression was evaluated using rabbit polyclonal Ab 2638 as described in the methods, and detected using a TexasRed conjugated secondary anti-rabbit Ab. Cells were counterstained with Hoechst 33342. The absence of paraquat exposure, or length of treatment with 150 µm paraquat is indicated above each panel. Detection of EBNA1-specific signal, Hoechst signal and the merge is also indicated. The scale bar represents a distance of 10 µM.

These experiments were conducted in an LCL, NOLA-1, immortalized using the B95-8 strain of EBV. In preliminary experiments, it was observed that while treatment of NOLA-1 with 1.4 µM menadione was toxic within 18 hours, treatment with 150 µM paraquat was not toxic for at least 72 hours. Therefore, NOLA-1 cells were treated with 150 µM paraquat. Aliquots of cells were removed at 48 and 72 hours to assay transcription from the *Bam*HI-Cp promoter by real-time PCR, the results of which are shown in Figure 8A. Within 48 hours of treatment with 150 µM paraquat, the level of *Bam*HI-Cp transcripts was observed to decrease to approximately 60% of the level observed in control cells. This decrease was significant, with a *p-*value of less than 0.01. After 72 hours of treatment, a further decrease in the level of *Bam*HI-Cp transcripts was observed, such that it was approximately 40% the level observed in control cells (*p*<0.01). Treatment with paraquat did not decrease the viability of cells as estimated by mitochondrial respiration for up to 72 hours (data not shown). In addition, a cell-cycle analysis indicated that paraquat treatment did not increase the fraction of sub-G1 cells, although paraquat-treated cells clearly accumulated in G0/G1 after 72 hours of exposure (Figure 8A). Staining with Annexin V and propidium iodide indicated a small increase in the numbers of doubly-positive necrotic cells, and annexin V-positive apoptotic cells as a consequence of paraquat treatment. However, we emphasize that the majority of cells remained doubly-negative (Figure 8B). Therefore, the decreases observed in *Bam*HI-Cp transcription do not result from cellular necrosis or apoptosis.

The decrease in *Bam*HI-Cp transcription was confirmed by examining protein levels for EBNA2 and LMP1 by immunoblot, along with the levels of EBNA1. This analysis is shown in Figure 8B. No significant decrease in EBNA1 protein levels were observed after 72 hours of exposure to paraquat, indicating that the decrease in *Bam*HI-Cp transcription does not result from a reduction in EBNA1. At the same time-point, an approximately 50% decrease in the levels of EBNA2 and LMP1 were observed. Both these proteins have substantially shorter half-lives than EBNA1 [69],[70],[71], and at least for EBNA2 the decreased protein level parallels the decrease in *Bam*HI-Cp transcription. The decrease in levels of LMP1 could either result from a diminution in transactivation by EBNA1, or by a reduction in the levels of EBNA2, as both proteins are known to transactivate the LMP1 promoter [11],[12],[13],[72],[73].

Finally, indirect immunofluorescence was used to determine whether oxidative stress altered the sub-cellular localization of EBNA1, as has been observed for other transactivators. This analysis is shown in Figure 8C, and indicates that although small changes in the nuclear localization of EBNA1 are observed even by 48 hours of exposure to paraquat, the majority of EBNA1 remains nuclear. During microscopy, mitotic figures were observed for approximately 5% of control cells, with punctate EBNA1 staining (two such cells are shown in Figure 8C). Such mitotic cells were rarely observed after even 48 hours of paraquat treatment, consistent with the observation that treated cells accumulate in G0/G1.

In summary, limited oxidative stress does not alter the levels or nuclear localization of EBNA1, but decreases *Bam*HI-Cp transcription, and reduces EBNA2 and LMP1 levels. Concurrent with these changes, treated cells accumulate in G0/G1.

## Discussion

At the onset of these studies, it was known that deletion of UR1 abolished EBNA1's ability to transactivate [10], and that UR1-deleted EBV failed to immortalize naïve B-cells [2]. We have dissected the mechanism by which UR1 functions for EBNA1 to transactivate, and observed that transactivation is subject to regulation by environmental conditions.

We have determined that the UR1 domain of EBNA1 coordinates zinc through two conserved, essential cysteines, and upon doing so self-associates. Point mutation of these cysteines eliminates the ability of EBNA1 to self-associate, coordinate zinc, or activate transcription. In addition, chelation of intracellular zinc severely impacts the ability of EBNA1 to activate transcription, and blocks the ability of UR1 to support self-association. Together these data provide the first evidence that a domain of EBNA1 necessary for transactivation coordinates zinc, and upon doing so, allows EBNA1 to self-associate.

Because short motifs containing two cysteines residues have been known to mediate heterotypic protein interactions, as exemplified by the interaction between the *lck* kinase and CD4 or CD8 [74], we explored the possibility that through zinc coordination UR1 permits EBNA1 to interact with another protein such as a cellular transcription co-activator. Indeed, it has been shown recently that the UR1 region of EBNA1 interacts with the cellular Brd4 protein, and interpreted that this interaction contributes to transactivation [43]. Our failure to find proteins such as Brd4 could either reflect their rarity, or that co-activators only associate with an array of EBNA1 bound to FR, a condition not met by our two-hybrid screen. However, we note, that co-activators were also not found in previous one-hybrid screens in which FR-bound EBNA1 was used as bait [42],[75]. In addition, while deletion of UR1 severely reduces transactivation, an EBNA1 derivative with multiple copies of UR1 did not activate transcription to higher levels than wild-type [76], as might be expected if UR1 functions by recruiting a transcription coactivator. Therefore, we propose that UR1 contributes structurally to EBNA1's ability to transactivate, when it is bound to FR. Such a structural contribution likely explains our observation that while the amino-terminal 450 a.a. of EBNA1 contains domains sufficient for transactivation, it does not squelch EBNA1's ability to transactivate. The inefficiency with which FLAG-tagged EBNA1(1-450)-E2DBD pulls down EBNA1 reinforces our belief that for intact EBNA1, self-association through UR1 is facilitated upon binding FR. An implication of the results obtained when comparing transactivation by EBNA1 and EBNA1(CC→SS) is that an intact UR1 domain is required for cooperative transactivation. Thus, cooperative association with an array of cognate binding sites by the DBD of EBNA1 does not solely underlie EBNA1's ability to activate transcription cooperatively.

The mechanisms for transactivation that we have considered are grounded in the prior observation that EBNA1 contains a self-interacting domain that maps to UR1 [16], and that several zinc-coordinating domains are known to self-associate through homotypic interactions. Examples of the latter include zinc-binding domains within the *Ikaros* family of proteins [29], the E7 protein of HPV18 [19], and the Tat protein of HIV-1 [77]. Studies on many transactivators indicate that homotypic interactions are often associated with structural changes in the template that facilitate transactivation. These include architectural proteins such as HMGA1a [28], cellular transcription factors such as p53, Sp1, and *Ikaros* [30],[32],[78], and the E2 transactivator of HPV [79],[80].

Using these observations as precedent, and the knowledge that for transactivation EBNA1 requires both UR1 and AT-hook domains, we propose two models by which UR1 contributes to transactivation. In the first model, it is speculated that UR1 mediates loop formation between EBNA1 bound to FR and as yet unidentified promoter proximal sites, and that such loops facilitate the association of EBNA1's AT-hooks with AT-rich sequences present at EBV promoters such as *Bam*HI-Cp. In the second model, which we believe is more consistent with our data, we propose that UR1 facilitates interactions between EBNA1 dimers bound to adjacent sites in FR to create a structured array of EBNA1 at FR. Further, that the structure presented by such an array facilitates the interaction of EBNA1's AT-hooks with promoter proximal AT-rich sequences, and the possible recruitment of co-activators.

The mechanism by which UR1 dimerizes suggests that transactivation can be regulated by the availability of zinc, and by modulating the ability of the two conserved cysteines in UR1 to participate in zinc coordination. Zinc levels are known to differ in various body compartments, and the function of immune cells is dependent on zinc [81],[82]. Therefore, it is possible that the site a latently-infected cell resides in affects the function of EBNA1 within that cell. Cellular zinc homeostasis is known to be regulated finely [83], and has recently been shown to be relevant to the pathogenesis of oncogenic papillomaviral infections, through the action of the viral E6 and E7 proteins on cellular zinc-binding proteins such as metallothioneins [84], and modulation of cellular zinc transporters by the viral E5 protein [85]. Cellular zinc availability directly affects the activity of the eukaryotic transactivators such as MTF-1 in human cells [86],[87], ZafA in *Aspergillus* [88], and Zap1 in *Saccharomyces* [89].

The oxidation status of cysteines affects the activities of many proteins that participate in gene expression including transcription factors and the SMN splicing complex [53],[55]. Such an effect is pronounced for zinc-binding proteins whose structure and function are altered by cysteine oxidation. Indeed, it is estimated that estrogen receptor isolated from approximately one-third of untreated ER-positive breast tumors is unable to bind its cognate binding site [52], a defect that can be reversed by treating cell extracts with reducing agents such as dithiothreitol [90]. For some zinc-finger proteins such as Egr-1, over-expression of the cellular redox mediator, Ref-1/APE1, and hypoxic conditions are shown to increase activity both by increasing the synthesis of Egr-1, and facilitating its nuclear translocation [91]. For the glucocorticoid receptor, redox modulates nuclear translocation in addition to DNA binding [92].

It is intriguing that the ability of EBNA1 to transactivate is modulated by intracellular redox, and that the activity of EBNA1 differs under normoxic and hypoxic conditions. B-cells latently infected by EBV exist as proliferating blasts in lymph nodes expressing all the latency-associated genes necessary for cell proliferation (latency III), and as a subset of quiescent memory B-cells in the periphery where the only viral protein expressed is EBNA1, and that too only when cells divide (latency 0/I) [62],[63]. This striking difference in phenotype correlates with epigenetic changes associated with viral promoters active during latency III and shutdown during latency I. There are prominent physiological differences between these two niches that latently-infected cells reside in. Sites like the spleen, thymus and lymph nodes are hypoxic with an effective oxygen level of between 2% and 5%. In contrast, the periphery is relatively oxygen-rich, with an effective oxygen level of approximately 10%. Studies conducted over the last 40 years have indicated that oxygen levels affect the behavior of B-cells; Mishell and Dutton [93] first showed that the proliferation and antibody production properties of murine splenic B-cells was augmented considerably under hypoxic conditions. There have been studies since then indicating that cytokine production properties of T-cells and B-cell differ considerably when the cells are grown under hypoxic conditions that mimic oxygen levels *in vivo*, or under normoxic conditions that typically prevail when cells are grown in culture [59],[94].

Exposure of a proliferating EBV-immortalized LCL to mild oxidative stress decreases transcription from the viral *Bam*HI-C promoter, consistent with observation made with transcription reporter plasmids in transformed cell-lines. This decrease in transcription manifests itself as decreased levels of the viral EBNA2 and LMP1 proteins, which are essential for the continued proliferation of EBV-immortalized cells [95],[96]. Treatment was not observed to affect LCL survival, although treated cells did accumulate in G0/G1. As such, there are parallels between these results, and those obtained with recombinant EBVs expressing conditional derivatives of EBNA2 and LMP1 (*ibid*). Conditional inactivation of EBNA2 after immortalization results in a rapid depletion of LMP1. Such cells exit the cell-cycle, and undergo necrosis [95]. It is likely that our treated cells do not undergo necrosis because EBNA2 and LMP1 are only partially depleted. Conditional inactivation of LMP1 causes newly-infected cells to become cell-cycle arrested, but survive quiescently [96]. It is known that the expression level of LMP1, as well as its sub-cellular localization can dramatically affect cellular physiology [97],[98],[99],[100]. Thus although we observe only a 50% decrease in the level of LMP1, this decrease may be sufficient to down-modulate mitogenic signals required for cell proliferation.

While it has been long known that hypoxic conditions alter the propagation and pathogenicity of bacterial, viral and eukaryotic pathogens, the molecular details elucidating these effects have emerged more recently. Redox controls the activity of the OxyR family of prokaryotic transactivators, such that changing culture conditions from anaerobic to aerobic induces the expression of OxyR-induced genes [101]. The Tat protein of HIV-1 induces apoptosis of naïve T-cells under normoxic conditions; yet, at physiological oxygen levels (hypoxic conditions), Tat stimulates T-cell proliferation, and primes them for infection by HIV-1 [102]. The ability of the papillomaviral E2 protein [103], and EBV's lytic transactivator BZLF1/Zta [104] to support replication is regulated by redox. Our results raise the intriguing possibility that in the severely hypoxic conditions prevalent in lymph nodes EBNA1 transactivates the expression of viral genes required for cell proliferation, but when such cells escape to the periphery, higher oxygen levels gradually diminish the ability of EBNA1 to transactivate while leaving intact its ability to maintain viral genomes. This model is consistent with the observation that EBNA1 with UR1 deleted continues to support EBV genome replication and partitioning, and is not inconsistent with the ability of EBV to efficiently immortalize B-cells grown *in vitro* under normoxic conditions. It is known that exposure of primary cells under hypoxic conditions to normoxia induces the expression of redox genes, particularly thioredoxin, and consistent with this, established EBV-positive lymphoma lines grown *in vitro* express considerably higher levels of thioredoxin than primary B-cells [105].

In summary, our studies provide mechanistic insights that underpin the ability of EBNA1 to self-associate and to activate transcription. Transactivation by EBNA1 relies on its ability to interact homotypically by coordinating zinc through two conserved cysteines. EBNA1's ability to transactivate is subject to regulation by the availability of zinc, and oxygen levels in the environment, implying a mechanism to modulate EBNA1's ability to transactivate without affecting its ability to stably maintain EBV episomes. The regulatory model proposed here for EBNA1 is likely applicable to proteins of other viral, prokaryotic, and eukaryotic pathogens that traffic between environmentally distinct niches within the body.

## Materials and Methods

### Effector and reporter plasmids

Plasmids expressing wild-type EBNA1, the EBNA1 DBD and HMGA1a-DBD have been described previously [6],[26],[106]. Similar plasmids were constructed to express EBNA1Δ(71-88), EBNA1(CC→SS), and UR1-DBD. The chimeric protein 3xF-EBNA1(1-450)-E2DBD was constructed by fusing a.a. 1–450 of EBNA1 fused to a.a. 239–410 of BPV-1 E2, with a 3xFLAG epitope tag at the amino-terminus of EBNA1. The chimeric protein DBD-VP16 was constructed by fusing the EBNA1 DBD to a.a.413–490 of HSV-1 VP16. For bimolecular fluorescence complementation, a.a. 61–90 of EBNA1 was fused independently to a.a. 155–238, and 2–154 of EYFP. FR-TKp-luciferase [3] reporter plasmid and *oriP*-*Bam*HI-Cp-luciferase reporter plasmid [26] were described previously. The 2xMME-TKp-luciferase reporter, was constructed by replacing FR in FR-TKp-luciferase with two copies of the BPV-1 MME [107]. The Ref-1/APE1 expression plasmid was a gift from Dr. Tadahide Izumi. Derivatives of the FR-TKp-luciferase reporter plasmids with one, three, five, seven, ten, and 20 binding sites have been described previously [3].

### Cell culture

Experiments were performed in C33a (HPV-negative) cervical cancer cells, BJAB (EBV-negative) Burkitt's lymphoma cells, and 293 human embryonic kidney epithelial cells, propagated and transfected as described previously [3],[5],[6]. NOLA-1 is a lymphoblastoid cell-line established from the peripheral B-cells of an HIV-1 negative donor by infection with B95-8 EBV, using established protocols [108]. Cells used for these experiments have been passaged fewer than fifteen times after an immortalized clone was obtained.

### Immunoblotting

Proteins were immunoblotted as described earlier [5],[6]. Rabbit polyclonal antibody K67.3 was used to detect EBNA1, and the M2 mouse monoclonal anti-FLAG antibody (Sigma, St. Louis, MO) was used to detect FLAG-tagged proteins. Goat polyclonal antibody 2824 was used to detect EBNA2, and rabbit polyclonal antibodies 2825/2826 were used to detect LMP1. Polyclonal antibodies against EBNA1, EBNA2 and LMP1 were obtained from Pacific Immunology using three antigenic peptides from each protein. Mouse monoclonal antibody (8226) against β-actin was purchased from Abcam (Cambridge, MA).

### Luciferase reporter assays

Reporter assays were performed as described earlier [3] using 500 ng of the reporter plasmid and 10 µg of the effector plasmid. Flow cytometry was used to normalize raw luciferase values to correct for the percent of live-transfected cells (PI-negative, GFP-positive) in each transfection. In experiments examining cooperative transactivation, 100 ng of each reporter plasmid was co-transfected with 1 µg of the effector plasmid and 500 ng of a GFP expression plasmid. Raw luciferase values were corrected for the percent of live-transfected cells. Cooperativity plots were generated by non-linear regression analysis fit to a sigmoidal dose-response curve using Prism (GraphPad Software, La Jolla, CA).

### Metal-ion chelation and supplementation

N, N, N′, N′-Tetrakis- (2-pyridylmethyl)-Ethylenediamine (TPEN) (Sigma, St Louis, MO) was used to chelate zinc. After transfection, cells were incubated with TPEN for 15 hours, following which they were analyzed to determine the percent of live-transfected cells, the cell-cycle profile, and luciferase activity. In supplementation experiments, the indicated metal salt was added to media already containing TPEN. Metals were added as solutions of the followings salts, zinc acetate, cadmium acetate, ferrous acetate, calcium chloride, manganese chloride, and magnesium sulfate. All salts were purchased at the highest purity available from Fisher Scientific, Pittsburgh, PA.

### Zinc^65^ blotting

Radioactive zinc blotting was done as described previously [17],[18],[19]. Briefly, synthetic peptides were obtained at >99% purity and resuspended in 50 mM Tris pH 7.4 at 10 mg/ml (Peptide 2.0, Chantilly, VA). The WT peptides corresponded to a.a 58–89 of wild type EBNA1 (WT) or the cysteine to serine mutant (M) described above. WT and M peptides were blotted on a PVDF membrane. Membranes were incubated with approximately 50 µCi of ^65^ZnCl_2_ (specific activity .0442 mCi/µg, Oak Ridge National Laboratory, Oak Ridge, TN) for 30 minutes at room temperature, washed and analyzed by phosphor-imaging. Membranes prepared in parallel, were stained with Amido-Black.

### Co-immunoprecipitation

293 cells were co-transfected with plasmids expressing EBNA1(1-450)-E2DBD, and EBNA1 or EBNA1(CC→SS). Co-immunoprecipitation was performed as described [5] using the M2 mouse monoclonal antibody conjugated to sepharose beads. Immunoprecipitates were detected using M2 anti-FLAG antibody (Sigma, St. Louis, MO), or K67.3 anti-EBNA1 antibody.

### Bimolecular fluorescence complementation (BiFC)

293 cells were co-transfected with plasmids expressing EYFP(2-154) and EYFP(155-38), or the corresponding UR1 fusions. Fixed cells were examined by fluorescence microscopy 48 hours post-transfection. In some experiments, the zinc chelator 1,10-phenanthroline (Fisher Scientific, Pittsburgh, PA) was added 24 hours prior to microscopy. To analyze the effect of redox, menadione (MP Biomedicals, Solon, OH) in various concentrations was added to the culture medium six hours post-transfection.

### Size exclusion chromatography

Size exclusion chromatography was performed using a Biosep-SEC-S2000 (600×30 mm) (Phenomenex, Torrance, CA) column on a Bio-Rad BioLogic Duo Flow system, to examine the ability of WT and M peptides to form dimers or higher order multimers. The column was equilibrated with a buffer consisting of 50 mM NaCl and 50 mM Tris (pH 7.5), and run in the same buffer at 0.8 ml/min (approximately 730 psi) for 50 minutes after sample injection. Molecular weights were calculated after the column was calibrated with Bio-Rad gel filtration standards ranging in size from 1.35 to 670 kD. Elution from the column was monitored by absorption at 280, 225, 215 and 205 nm using a QuadTech inline detector. For calculation, the log of the molecular weights of standards was plotted against retention time. The method was found to be as accurate as using the ratio of the elution volume to the void volume, and was validated by calculating the molecular weight of purified ubiquitin and cytochrome C based on their retention times. When the effect of zinc on multimerization was tested, it was added to the peptide at a final concentration of 1 mM.

### Redox experiments

C33a cells were transfected as described [3] with plasmids as depicted in Figure 6. Six hours post-transfection, cells were split and treated with menadione or paraquat for 18 hours following which they were analyzed for their cell-cycle profile, and luciferase activity.

### Hypoxia experiments

C33a cells were transfected as described [3]. Six hours post-transfection, cells were split, and grown under normoxic conditions, or hypoxic conditions. For hypoxic conditions, cells were grown in a sealed modular incubation chamber (Billups-Rothenberg, Inc, Del Mar, CA) placed at 37^o^C. Prior to sealing, the chamber was flushed with a mixture of 4%O_2_, 5%CO_2_ and 91%N_2_ gas (AirGas, Theodore, AL) for five minutes. Culture media was replaced with fresh media every 24 hours that was pre-equilibrated to normoxic or hypoxic conditions, at which time the chamber was re-flushed and re-sealed. No significant differences in doubling rate were observed between normoxic and hypoxic conditions. Southern blots were performed as described [3] using probes specific for *oriP*-*Bam*HI-Cp-luciferase.

### Flow cytometry analyses

Apoptosis and necrosis was evaluated by flow cytometry using the Annexin V-FITC apoptosis detection kit (EMD Biosciences, Gibbstown, NJ) as per the manufacturers protocol, and examined using a FACSCalibur flow cytometer. Cell-cycle analysis was performed after PI-staining of fixed cells using a FACSCalibur.

### Real time PCR

Total cellular RNA was extracted using TRI Reagent (Ambion, Austin, TX), following which cDNA was prepared using TaqMan reverse transcriptase reagents (Applied Biosystems, Foster City, CA). Real time PCR was performed using the ABsolute blue QPCR Rox mix (Thermo Fisher, Germantown, PA) on an ABI 7300 real time PCR system (Applied Biosystems, Foster City, CA). The primer and probe sequences used for the detection of *Bam*HI-Cp transcripts and the endogenous control GAPDH mRNA have been described [109]. Relative quantitation was performed by the dCt method, and is expressed relative to the level of normalized *Bam*HI-Cp transcripts detected in control cells.

### Immunofluorescence microscopy

Control or treated NOLA-1 cells were examined by immunofluorescence using microscopy conditions described previously [76]. Rabbit polyclonal antibodies 2638 or K67.3 was used to detect EBNA1 with a TexasRed conjugated anti-rabbit secondary antibody (Jackson ImmunoResearch, West Grove, PA).

### Statistical analysis

All the statistical analysis where indicated was performed using MSTAT version 5 (N, Drinkwater, McArdle Laboratory for Cancer Research, University of Wisconsin Medical School). The Wilcoxon rank sum test was used for pair-wise comparisons.

## Supporting Information

Figure S1 Expression of the EBNA1 derivatives analyzed in Figure 1B. Extracts from 5×10^5^ transfected C33a cells were separated by SDS-PAGE electrophoresis, and examined by immunoblot as described in the Materials and Methods section. Rabbit polyclonal antisera (K67.3), raised against the DBD, was used as the primary antibody. The arrowheads indicate the migration of pre-stained markers of known molecular weights.
(2.32 MB TIF)

Figure S2 Expression of the EBNA1 and EBNA1(CC→SS) in transfected C33a cells. Extracts from 5×10^5^ cells were separated by SDS-PAGE electrophoresis, and examined by immunoblot as described in the Materials and Methods section, using K67.3 as the primary antibody. The arrowheads indicate the migration of pre-stained markers of known molecular weights.
(0.62 MB TIF)

Figure S3 The addition of zinc acetate reverses inhibition of transactivation caused by TPEN. C33a cells were co-transfected with the FR-TKp-Luciferase reporter plasmid, an EBNA1 expression plasmid, and a CMV-EGFP expression plasmid. Transfected cells were treated with 5 µM TPEN at the time of transfection. 15 hours post-TPEN addition, the indicated amounts of Zn(CH_3_COO)_2_ was added to the cells for an additional 15 hours. Cells were harvested and analyzed by flow cytometry to determine the level of lice-transfected cells, followed by determination of luciferase activity. The relative activation is shown in the grey bars, and is expressed as a percent of the luciferase activity observed in the untreated sample. The asterisks indicate significant increases (p<0.05 by Wilcoxon rank-sum test) in luciferase level upon addition of Zn(CH_3_COO)_2_ relative to addition of TPEN alone. The open bars indicate the percent of live EGFP-positive cells at each concentration of TPEN and Zn(CH_3_COO)_2_.
(0.31 MB TIF)

Figure S4 EBNA1(1-450)-E2DBD activates transcription from the 2xMME-TKp-Luciferase reporter. C33a cells were co-transfected with the 2xMME-TKp-Luciferase reporter plasmid, empty vector pcDNA3 or the EBNA1-E2DBD expression plasmid. Cells were harvested at 48 hours post-transfection, normalized by flow cytometry for the number of live-transfected cells, and analyzed for luciferase activity, which is expressed as fold activation relative to pcDNA3.
(0.22 MB TIF)

Figure S5 TPEN inhibits activation of 2xMME-TKp-luciferase by 3xF-EBNA1(1-450)-E2DBD. C33a cells were co-transfected with the 2xMME-TKp-Luciferase reporter plasmid, and the 3xF-EBNA1(1-450)-E2DBD expression plasmid. Transfected cells were treated with 5 µM TPEN for 15 hours and then analyzed, or with 5 µM TPEN for 15 hours followed by the addition of 5 µM Zn(CH_3_COO)_2_ for 15 additional hours prior to analysis. At harvest cells were analyzed by flow cytometry to determine the fraction of live-transfected cells, followed by assays for luciferase activity. The grey bars in the graph represent luciferase activity, which is expressed as a function of the luciferase activity observed in the absence of TPEN treatment 15 hours post-transfection.
(0.26 MB TIF)

Figure S6 Paraquat reduces the ability of EBNA1 to transactivate FR-TKp-Luciferase. C33a cells were co-transfected the FR-TKp-Luciferase reporter plasmid, and an EBNA1-expression plasmid. Cells were treated with the indicated levels of paraquat six hours post-transfection, and harvested 18 hours later. For cell-cycle analysis, an aliquot of cells was fixed and then PI-stained. The rest of the cells were processed to determine luciferase activity, which is expressed as a percent of the luciferase activity observed in the absence of paraquat treatment, The cell-cycle profiles of paraquat-treated and control cells were obtained for one experiment, and are shown below the graph.
(0.33 MB TIF)

Figure S7 Menadione does not decrease transactivation by DBD-VP16. C33a cells were co-transfected with the FR-TKp-Luciferase reporter plasmid and 2 µg of the DBD-VP16 expression plasmid. Cells were split six hours post-transfection, at which time half were treated with 1.4 µM menadione for 18 hours. Luciferase levels were determined 24 hours post-transfection, and are expressed as a percent of the transactivation observed in the untreated cells.
(0.21 MB TIF)

Figure S8 Over-expression of Ref-1/APE1 ameliorates the effect of paraquat on EBNA1 mediated transactivation. C33a cells were transfected with an EBNA1 expression plasmid, and either 2 µg of a Ref-1/APE1 expression plasmid or empty vector control plasmid in addition to the FR-TKp-luciferase reporter plasmid. Six hours post-transfection, the cells were split and half the cells were treated with 150 µM of paraquat. Luciferase levels were evaluated 24 hours post-transfection.
(0.29 MB TIF)
