# Supplementary figures and images for: Zinc Coordination Is Required for and Regulates Transcription Activation by Epstein-Barr Nuclear Antigen 1
Source: PLoS Pathog. 2009 Jun 12;5(6):e1000469. doi: 10.1371/journal.ppat.1000469 (PMC2690687; doi:10.1371/journal.ppat.1000469)

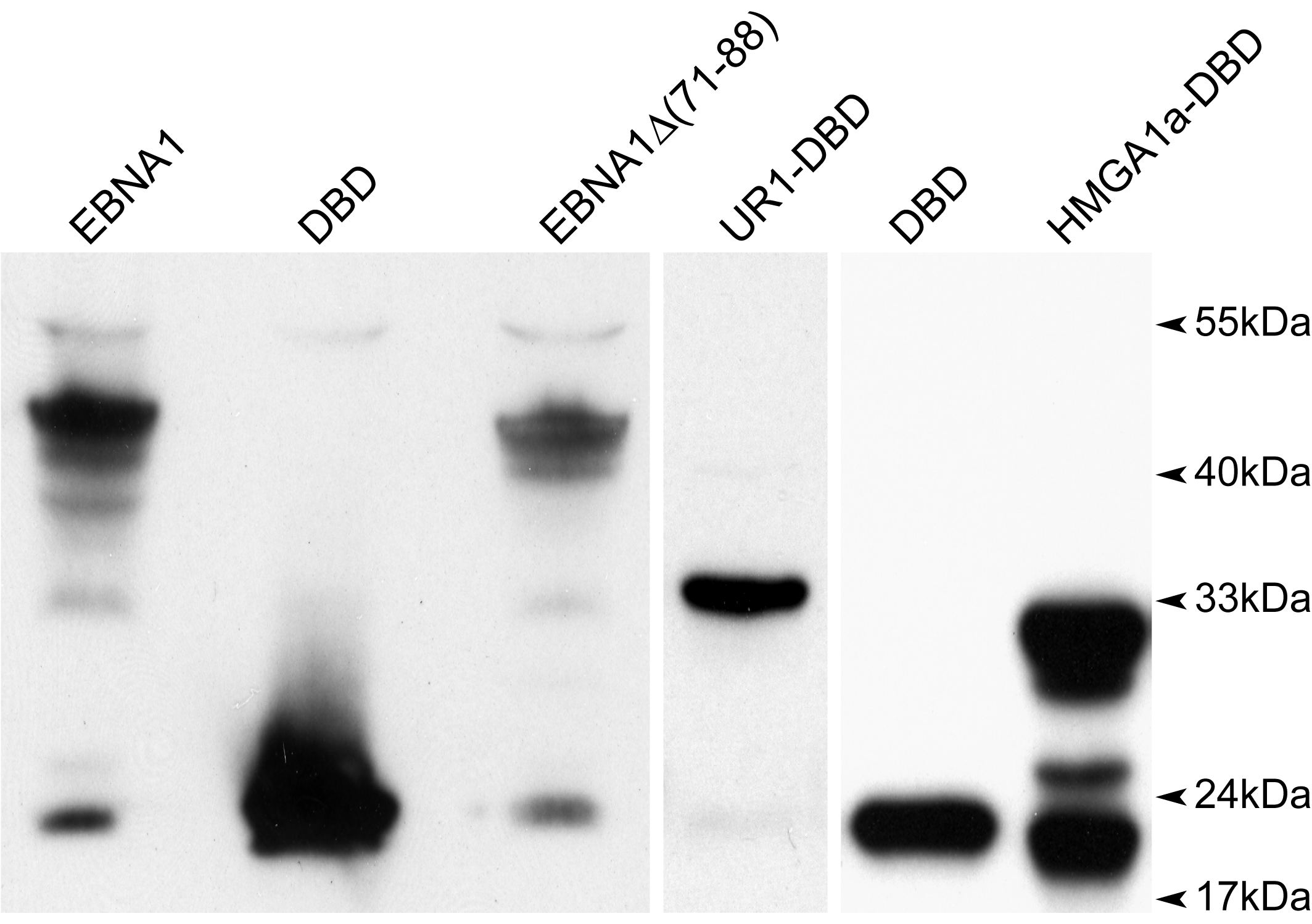

Supplement: Figure S1 — Expression of the EBNA1 derivatives analyzed in Figure 1B. Extracts from 5×10^5^ transfected C33a cells were separated by SDS-PAGE electrophoresis, and examined by immunoblot as described in the Materials and Methods section. Rabbit polyclonal antisera (K67.3), raised against the DBD, was used as the primary antibody. The arrowheads indicate the migration of pre-stained markers of known molecular weights. (2.32 MB TIF) [file ppat.1000469.s001.tif]

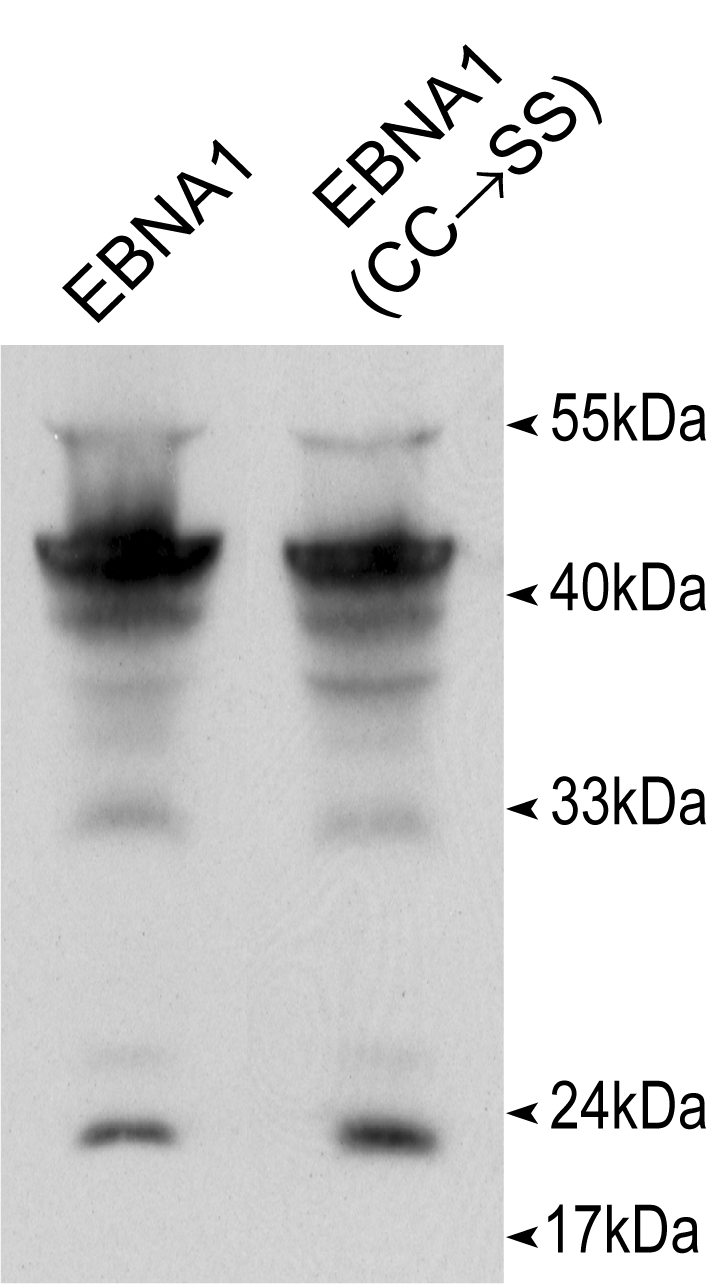

Supplement: Figure S2 — Expression of the EBNA1 and EBNA1(CC→SS) in transfected C33a cells. Extracts from 5×10^5^ cells were separated by SDS-PAGE electrophoresis, and examined by immunoblot as described in the Materials and Methods section, using K67.3 as the primary antibody. The arrowheads indicate the migration of pre-stained markers of known molecular weights. (0.62 MB TIF) [file ppat.1000469.s002.tif]

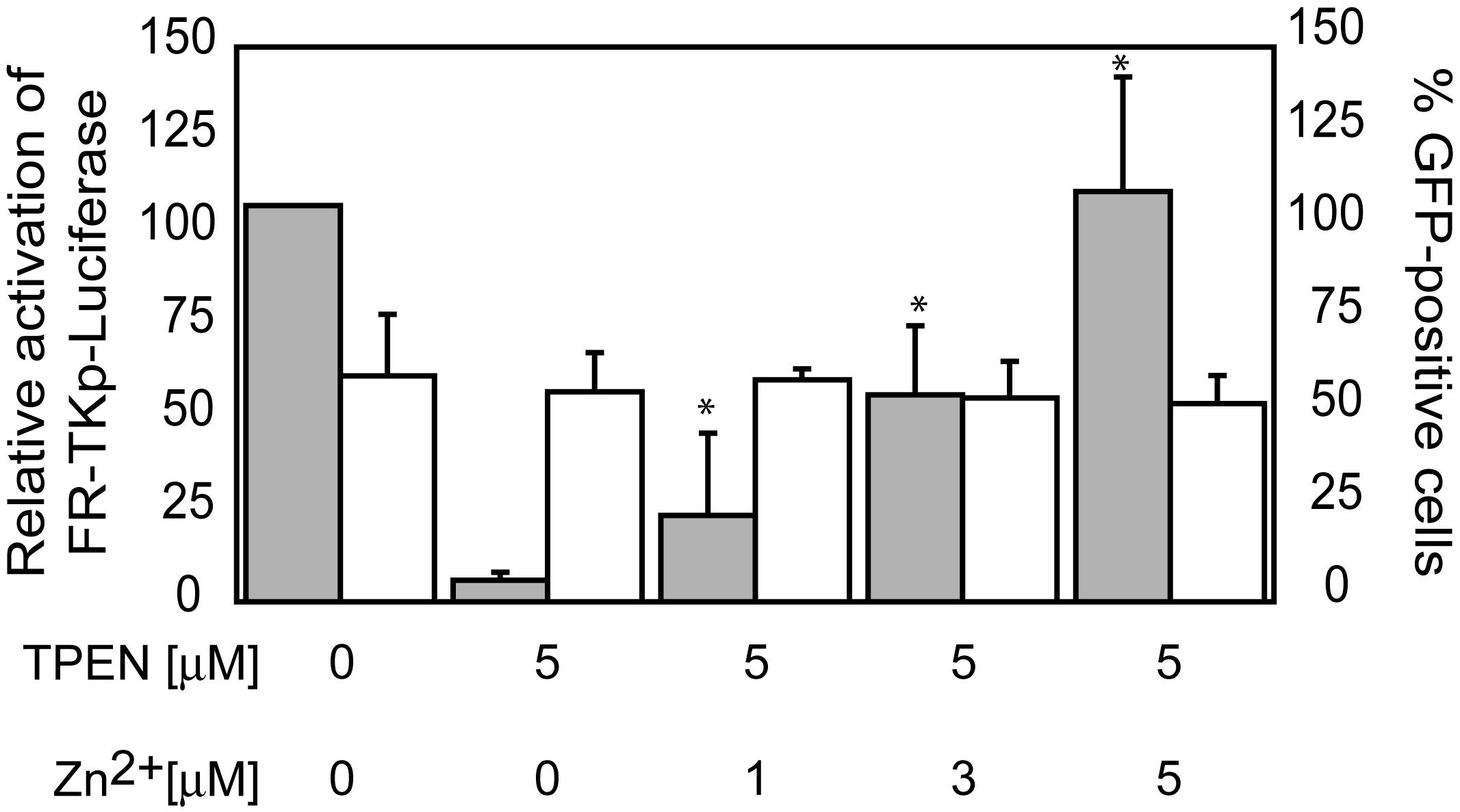

Supplement: Figure S3 — The addition of zinc acetate reverses inhibition of transactivation caused by TPEN. C33a cells were co-transfected with the FR-TKp-Luciferase reporter plasmid, an EBNA1 expression plasmid, and a CMV-EGFP expression plasmid. Transfected cells were treated with 5 µM TPEN at the time of transfection. 15 hours post-TPEN addition, the indicated amounts of Zn(CH3COO)2 was added to the cells for an additional 15 hours. Cells were harvested and analyzed by flow cytometry to determine the level of lice-transfected cells, followed by determination of luciferase activity. The relative activation is shown in the grey bars, and is expressed as a percent of the luciferase activity observed in the untreated sample. The asterisks indicate significant increases (p<0.05 by Wilcoxon rank-sum test) in luciferase level upon addition of Zn(CH3COO)2 relative to addition of TPEN alone. The open bars indicate the percent of live EGFP-positive cells at each concentration of TPEN and Zn(CH3COO)2. (0.31 MB TIF) [file ppat.1000469.s003.tif]

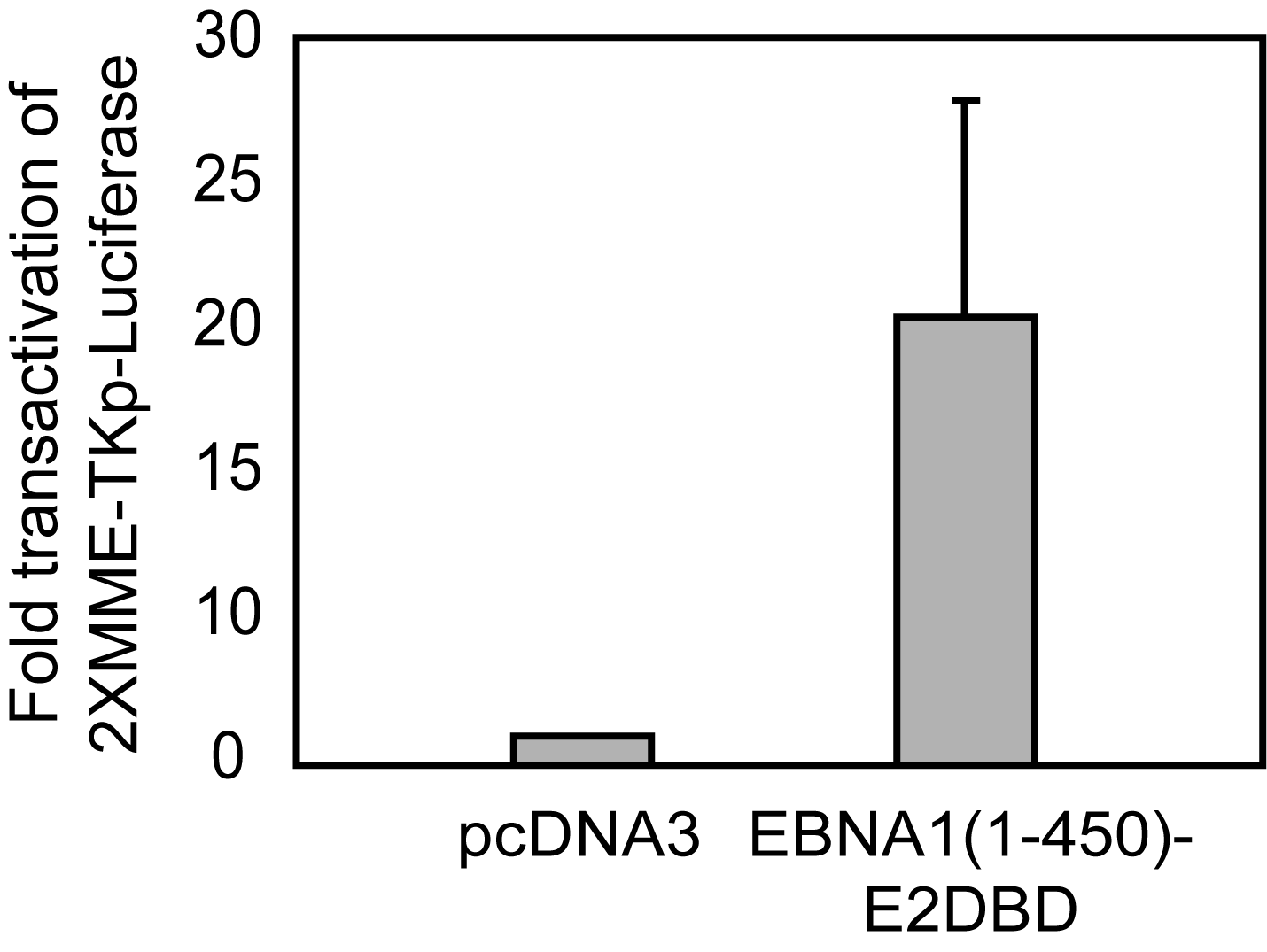

Supplement: Figure S4 — EBNA1(1-450)-E2DBD activates transcription from the 2xMME-TKp-Luciferase reporter. C33a cells were co-transfected with the 2xMME-TKp-Luciferase reporter plasmid, empty vector pcDNA3 or the EBNA1-E2DBD expression plasmid. Cells were harvested at 48 hours post-transfection, normalized by flow cytometry for the number of live-transfected cells, and analyzed for luciferase activity, which is expressed as fold activation relative to pcDNA3. (0.22 MB TIF) [file ppat.1000469.s004.tif]

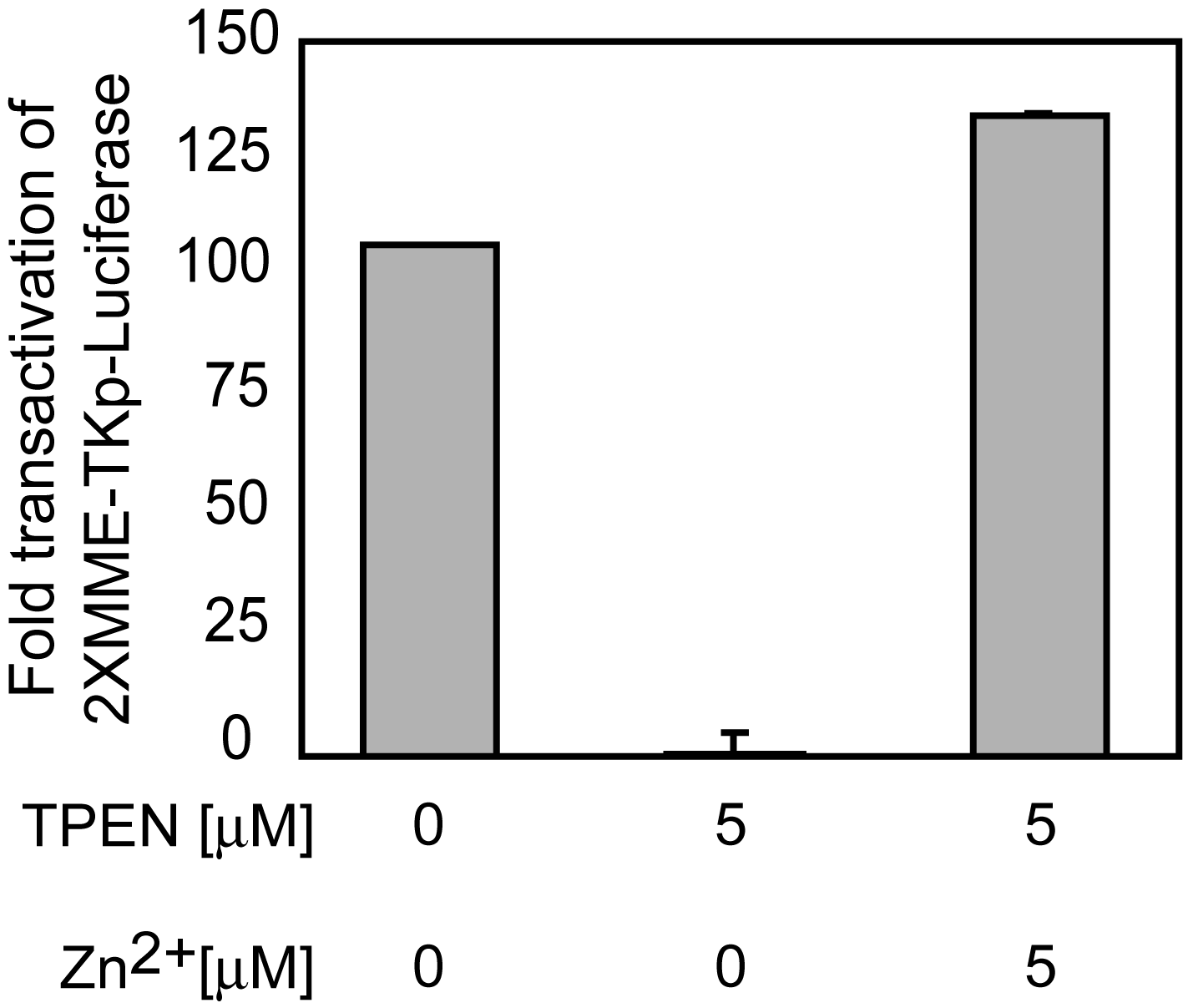

Supplement: Figure S5 — TPEN inhibits activation of 2xMME-TKp-luciferase by 3xF-EBNA1(1-450)-E2DBD. C33a cells were co-transfected with the 2xMME-TKp-Luciferase reporter plasmid, and the 3xF-EBNA1(1-450)-E2DBD expression plasmid. Transfected cells were treated with 5 µM TPEN for 15 hours and then analyzed, or with 5 µM TPEN for 15 hours followed by the addition of 5 µM Zn(CH3COO)2 for 15 additional hours prior to analysis. At harvest cells were analyzed by flow cytometry to determine the fraction of live-transfected cells, followed by assays for luciferase activity. The grey bars in the graph represent luciferase activity, which is expressed as a function of the luciferase activity observed in the absence of TPEN treatment 15 hours post-transfection. (0.26 MB TIF) [file ppat.1000469.s005.tif]

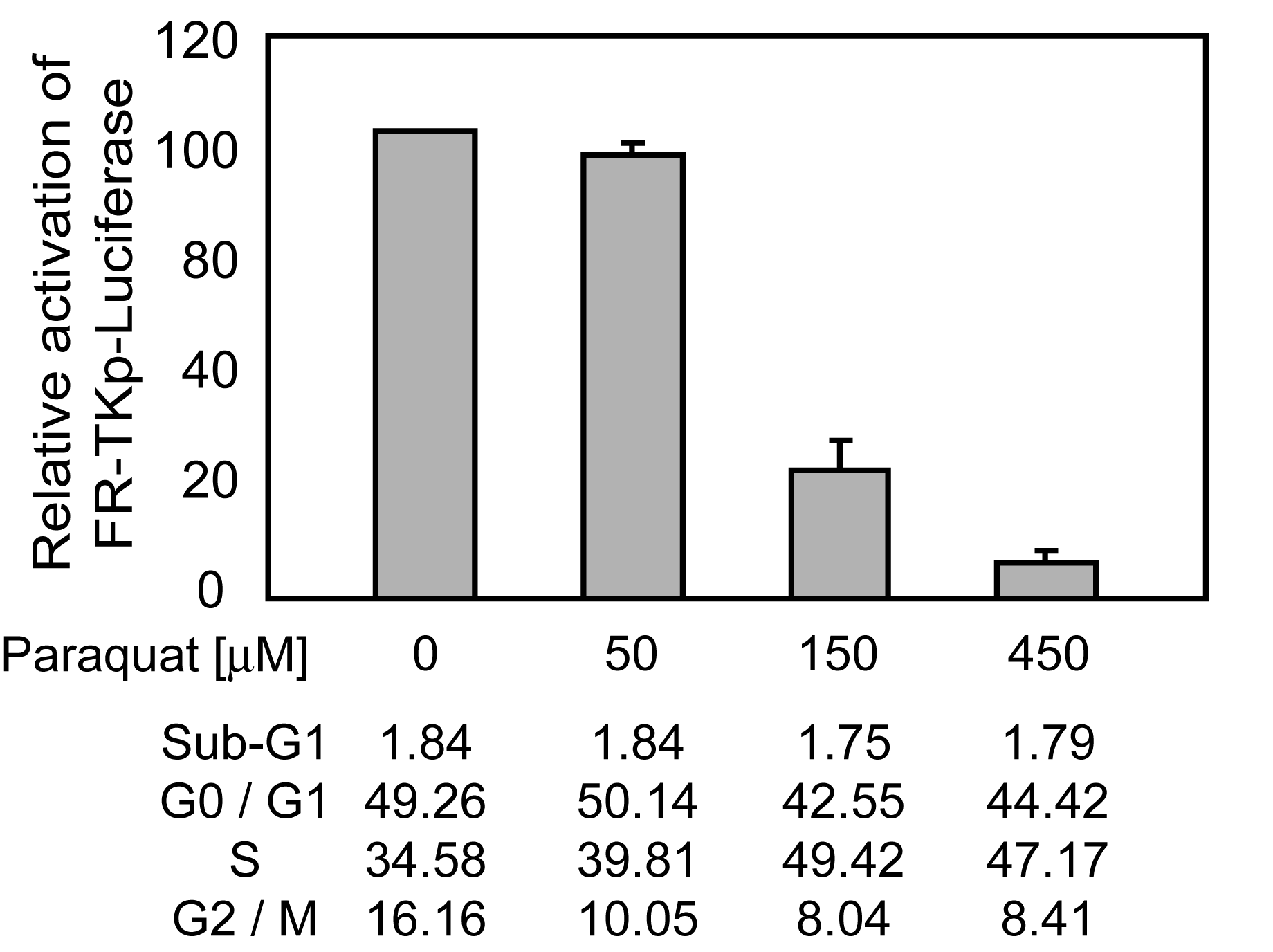

Supplement: Figure S6 — Paraquat reduces the ability of EBNA1 to transactivate FR-TKp-Luciferase. C33a cells were co-transfected the FR-TKp-Luciferase reporter plasmid, and an EBNA1-expression plasmid. Cells were treated with the indicated levels of paraquat six hours post-transfection, and harvested 18 hours later. For cell-cycle analysis, an aliquot of cells was fixed and then PI-stained. The rest of the cells were processed to determine luciferase activity, which is expressed as a percent of the luciferase activity observed in the absence of paraquat treatment, The cell-cycle profiles of paraquat-treated and control cells were obtained for one experiment, and are shown below the graph. (0.33 MB TIF) [file ppat.1000469.s006.tif]

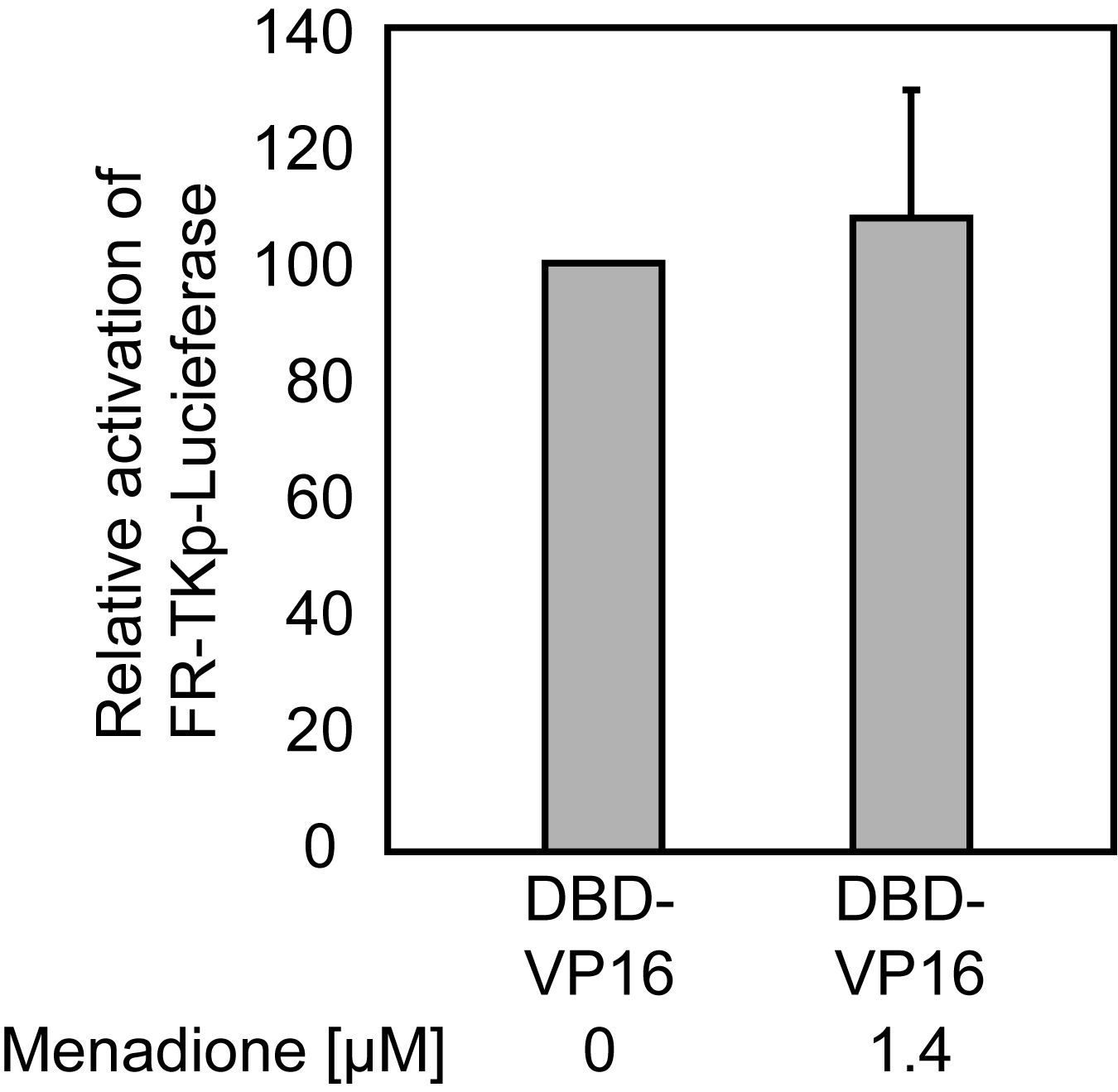

Supplement: Figure S7 — Menadione does not decrease transactivation by DBD-VP16. C33a cells were co-transfected with the FR-TKp-Luciferase reporter plasmid and 2 µg of the DBD-VP16 expression plasmid. Cells were split six hours post-transfection, at which time half were treated with 1.4 µM menadione for 18 hours. Luciferase levels were determined 24 hours post-transfection, and are expressed as a percent of the transactivation observed in the untreated cells. (0.21 MB TIF) [file ppat.1000469.s007.tif]

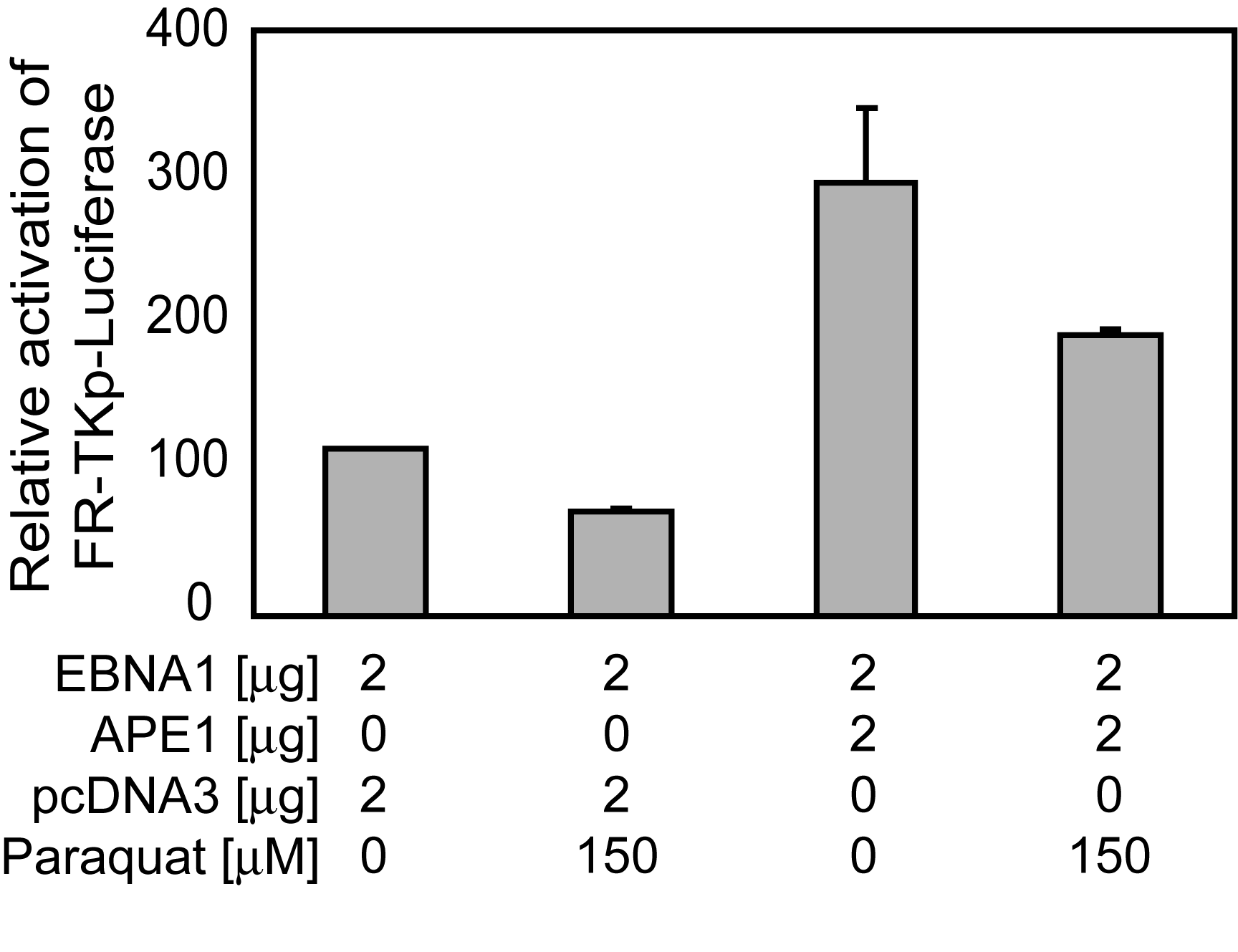

Supplement: Figure S8 — Over-expression of Ref-1/APE1 ameliorates the effect of paraquat on EBNA1 mediated transactivation. C33a cells were transfected with an EBNA1 expression plasmid, and either 2 µg of a Ref-1/APE1 expression plasmid or empty vector control plasmid in addition to the FR-TKp-luciferase reporter plasmid. Six hours post-transfection, the cells were split and half the cells were treated with 150 µM of paraquat. Luciferase levels were evaluated 24 hours post-transfection. (0.29 MB TIF) [file ppat.1000469.s008.tif]
